# Apolipoprotein E4 effects on topological brain network organization in mild cognitive impairment

**DOI:** 10.1038/s41598-020-80909-7

**Published:** 2021-01-12

**Authors:** Gretel Sanabria-Diaz, Lester Melie-Garcia, Bogdan Draganski, Jean-Francois Demonet, Ferath Kherif

**Affiliations:** 1 Laboratoire de Recherche en Neuroimagerie (LREN), Département des neurosciences cliniques, Centre Hospitalier Universitaire Vaudois (CHUV), Mont Paisible 16, 1011 Lausanne, Switzerland; 2 Leenaards Memory Center, Lausanne University Hospital (CHUV), Lausanne, Switzerland

**Keywords:** Alzheimer's disease, Network models, Genetic predisposition to disease

## Abstract

The Apolipoprotein E isoform E4 (ApoE4) is consistently associated with an elevated risk of developing late-onset Alzheimer’s Disease (AD); however, less is known about the potential genetic modulation of the brain networks organization during prodromal stages like Mild Cognitive Impairment (MCI). To investigate this issue during this critical stage, we used a dataset with a cross-sectional sample of 253 MCI patients divided into ApoE4*-*positive (‛Carriers’) and ApoE4*-*negative (‘non-Carriers’). We estimated the cortical thickness (CT) from high-resolution T1-weighted structural magnetic images to calculate the correlation among anatomical regions across subjects and build the CT covariance networks (*CT-Nets*). The topological properties of *CT-Nets* were described through the graph theory approach. Specifically, our results showed a significant decrease in characteristic path length, clustering-index, local efficiency, global connectivity, modularity, and increased global efficiency for Carriers compared to non-Carriers. Overall, we found that ApoE4 in MCI shaped the topological organization of *CT-Nets*. Our results suggest that in the MCI stage, the ApoE4 disrupting the CT correlation between regions may be due to adaptive mechanisms to sustain the information transmission across distant brain regions to maintain the cognitive and behavioral abilities before the occurrence of the most severe symptoms.

## Introduction

Late-onset Alzheimer’s Disease (AD) is a degenerative brain disease and the most common form of dementia in late-life, affecting millions of people worldwide^1^. Because of the lack of treatment, identifying causal risk factors at the early stages is paramount in clinical investigation. Most of the research is focusing its attention on the Mild Cognitive Impairment (MCI) stage. MCI is considered an intermediate phase between normal aging and AD. It is mainly characterized by a decline in cognitive abilities that do not interfere with daily functioning^2^. These patients are at increased risk of developing AD or another dementia^1^. Epidemiological research suggests an estimated 40% to 60% of MCI individuals aged 58 years and older have underlying AD pathology^3,4^.

Nevertheless, MCI does not always lead to dementia; some patients remain stable or revert to a normal state while other progress to different brain pathologies. This clinical variability is based on the interplay between physiological, environmental, and genetic factors as part of the disease multifactorial etiology^5,6^. In those MCI cases destined to evolve to Alzheimer's Disease, this “window” is an opportunity to develop biomarkers that help to identify etiology and predict progression.

Our study is motivated by the fact that the Apolipoprotein E isoform E4 (ApoE4) is the best-established genetic risk factor for AD^7^. Among MCI ApoE4 Carriers, previous studies have reported an increased risk of developing AD, a younger mean age of onset and more rapid cognitive decline than non-Carriers^8^. Likewise, in MCI, the prevalence of this genotype is substantially higher than in control individuals^9^. The ApoE4 mechanisms in AD's pathogenesis are not entirely understood but have been related to amyloid-β-dependent and independent pathways^10^. Although the amount of evidence linking ApoE4 with cognitive deficits, morphological, structural, and functional brain alterations during AD progression^11,12^ at this point, it is still unclear how this genetic risk factor impairs the brain networks organization.

Our study's second motivation is based on previous research supporting the idea of AD being a disconnection syndrome, which disrupts higher-order neuronal networks^13^. In this context, using a network-based approach is critical to understand brain alterations and cognitive deficits during the disease progression. One feasible mathematical approach to elucidate the AD impact on brain networks is the graph formalism^14^. In graph theory terms, our brain is studied as a model to describe some essential elements -nodes- (brain regions) and the relationship between them (edges). Afterward, the brain complex covariance patterns are translated into global and regional graph metrics^15^. During the last decade, the graph analysis has been applied to characterize the brain structural covariance in AD and MCI^16^. It is based on the phenomenon that regions correlated in morphometric descriptors (i.e., cortical thickness) are often part of the same brain system that subserve specific behavioral and cognitive functions^17^. The mechanisms underlying these coordinated patterns seem to be related to mutually trophic effects, common pathological vulnerabilities, and genetic factors^16^.

Following this modeling approach, studies using different neuroimaging modalities have shown aberrant brain network properties in AD, MCI, and preclinical states^18^. They revealed disease-related network alterations such as a loss of balance between segregation and integration of information (small-world attribute) and redistribution of regions considered central for the information flux over the network (hub regions)^19–23^. Additionally, Alzheimer's patients show decreased long-distance-interhemispheric correlations- and increased correlations between brain regions targeted by the disease^18,19,23^. These disruptions could reflect that the whole-brain network is more segregated and less integrated during AD progression than in healthy individuals. Despite such findings, the ApoE4 risk factor's inclusion has been scarce and limited mostly to healthy aging subjects and AD patients^24–28^.

There is only a handful of investigations on the ApoE4 effects on topological brain networks organizations in MCI. Two studies using resting-state Functional Magnetic Imaging (rs-fMRI) and diffusion weight imaging (DWI) compared MCI Carrier and non-Carriers groups^29,30^. In both cases, the network analysis showed specific aberrant patterns in MCI Carriers. Yao and colleagues^31^ reported for the first time differences between Carriers and non-Carriers based on metabolic covariance networks using Fluorodeoxyglucose Positron Emission Tomography (FDG-PET). Carriers were found to have lower clustering index and disruptive long-distance interregional correlations.

Nevertheless, ApoE4*-*related effects on the structural covariance network topology have not yet been fully explored in MCI. Such work is necessary to clarify how the genetic risks mediate and constrain the covariance patterns and the phenotypic expression in MCI. The identification of these subtle alterations at the network level may help detect, at earlier stages, the risk of AD progression in MCI ApoE4 Carriers compared to other disease-related markers like atrophy.

Precisely, we focus on the ApoE4*-*related modulation of the topological organization of cortical thickness covariance brain networks in MCI through structural MRI (sMRI) and graph-theory approach. We examine different features of the structural brain topology: (1) regional cortical thickness, (2) global network attributes (clustering index, characteristic path length, local and global efficiency, global connectivity, and homologous region connectivity) (3) nodal properties (normalized betweenness centrality, hubs) (4) network community detection (modularity) and resilience to insults (target attack). We hypothesize that ApoE4 is related to both local and global network properties changes in MCI.

## Materials and methods

### Subjects

Data used in the preparation of this article were obtained from the Alzheimer's Disease Neuroimaging Initiative (ADNI) database (adni.loni.usc.edu). The ADNI was launched in 2003 as a public–private partnership, led by Principal Investigator Michael W. Weiner, MD. ADNI's primary objective has been to test whether serial magnetic resonance imaging, positron emission tomography, other biological markers, and clinical and neuropsychological assessment can be combined to measure the progression of MCI and early Alzheimer’s Disease. For up-to-date information about ADNI, including Police and Procedures, see www.adni-info.org.

In the present study, 253 MCI participants with baseline T1-weighted structural magnetic resonance images were selected and downloaded from the USC's Laboratory of Neuroimaging ADNI (http://www.loni.ucla.edu/ADNI/).

The inclusion criteria were as follows: Mini-Mental-State-Examination (MMSE) scores between 24 and 30 (inclusive), a memory complaint, objective memory loss measured by education adjusted scores on the Wechsler Memory Scale Logical Memory II, a Clinical Dementia Rating (CDR) of 0.5, and absence of significant levels of impairment in other cognitive domains, essentially preserved activities of daily living and an absence of dementia.

Exclusion criteria included: (1) the presence of a major depressive disorder or significant symptoms of depression; (2) modified Hachinski ischemia score greater than 5; (3) significant neurological or psychiatric illness; (4) use of antidepressant drugs with anticholinergic side effects; (5) high dose of neuroleptics, chronic sedatives, hypnotics, antiparkinsonian medication, and use of narcotic analgesics. Detail about inclusion/exclusion criterium can be found in http://adni.loni.usc.edu/wp-content/themes/freshnewa-dev-v2/clinical/ADNI-1_Protocol.pdf)^32^.

The MCI group was stratified into those with one ApoE4 allele (Carriers) and those without (non-Carriers). ApoE genotyping details can be accessed at http://adni.loni.usc.edu/data-samples/clinical-data/^33^. Participants with one or more E2 allele(s) were excluded from this study due to the allele's possible protective effects^34^.

The subjects also met the following criteria: anatomical study acquired in a 1.5 T MRI-scanner, right-handedness, high sMRI image quality. For biomarker's measurements (Cerebrospinal fluid) characteristics, see Supplementary Information.

### Ethical statements

As per ADNI protocols, all procedures performed in studies involving human participants were under the institutional national research committee's ethical standards and the 1964 Helsinki declaration and its later amendments or comparable ethical standards. More details can be found at adni.loni.usc.edu. Participants were studied under ADNI protocols approved by the Institutional Review Board (IRB) at each recruitment site, and written informed consent was obtained from all subjects prior to enrollment. A listing of sites with named Site Investigators can be found online at http://adni.loni.usc.edu/wp-content/themes/freshnews-dev-v2/documents/policy/ADNI_Acknowledgement_List%205-29-18.pdf.

### Data acquisition and preprocessing

Preprocessed versions of the 253 baselines T1-weighted MRI scans were downloaded. Further details are available in the ADNI-MRI technical procedures manual (http://adni.loni.usc.edu/methods/documents/MRI protocols). Preprocessing steps can be found elsewhere^35–37^.

### Computation of mean cortical thickness matrices

**Figure 1:**
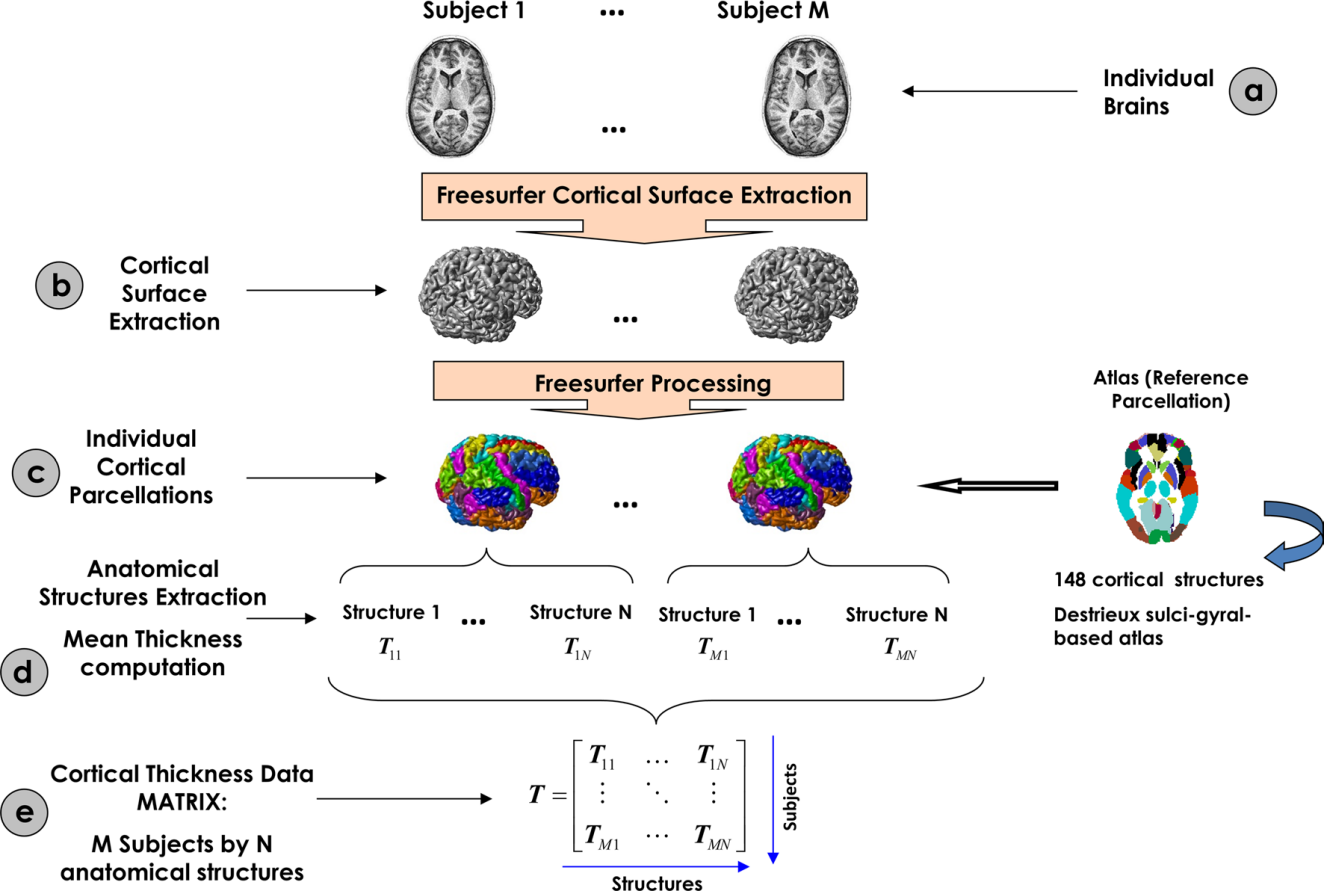
Flowchart of the cortical thickness matrix construction. (**a**) Representation of the M individual anatomic MRI images. (**b**) During FreeSurfer processing, the cortical surface of the M subjects was extracted. (**c**) Using the FreeSurfer toolbox, the cortical surfaces were labeled using a reference atlas. (**d**) The cortical thickness for each structure was calculated as the mean thickness of all vertices defined as belongs to that structure. (**e**) All mean thickness values for all structures and subjects were organized in an array denoted by T of M rows by N columns.

Cortical reconstruction and volumetric segmentation were performed using the *Freesurfer* analysis software suite with default settings (http://adni.loni.usc.edu/methods/documents/MRI protocols). The technical details of these procedures have been previously described^38^. *FreeSurfer* provides the cerebral cortex's parcellation based on Destrieux sulci-gyral-based atlas^39^ and the mean cortical thickness for each cortical structure. We used these outputs to construct our data matrices for each group. The number of rows corresponds to the number of subjects, while the number of columns corresponds to the number of structures (Fig. 1).

### Cortical thickness network construction

We defined a connection as statistical associations in cortical thickness between each pair of brain regions for a parcellation scheme of N = 148 anatomical structures (subcortical gray nuclei were excluded) (Supplementary Table S1 online). The synchronized covariations in cortical thickness between two regions were computed using Pearson's correlation coefficient across subjects. Thus, the interregional correlation matrix (N × N, N is the number of brain regions) of such connections was obtained using all pairs of anatomical structures*.* Self-connections were excluded implying zeros in the diagonal of the symmetric matrix. It is essential to point out that a partial correlation analysis could not be used in our case because the sample size was not large enough for a robust estimation of this measure.

Before the correlation analysis, a linear regression was performed at every region to remove the effects of age, gender, age-gender interaction, and cerebral mean cortical thickness.

In the next step, we obtained for each MCI group Nboot = 2000 bootstrap samples of the connectivity matrix by selecting a random subset of subjects with replacement using the classical bootstrapping procedure described in^40^.

**Figure 2:**
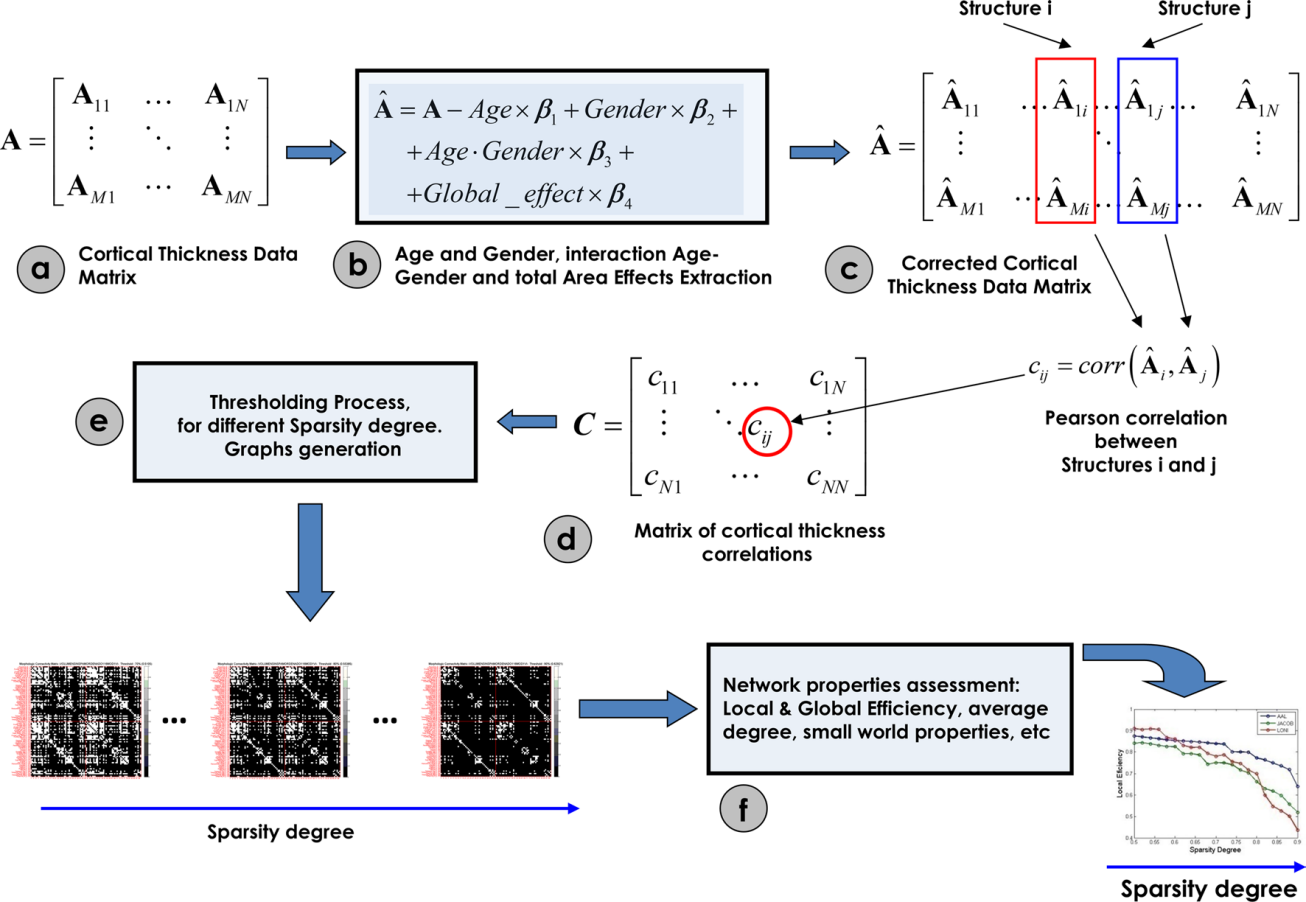
Steps for assessing the networks of cortical thickness covariance. (**a**) Matrix of the morphometric descriptor (cortical thickness) for the Destrieux parcellation. (**b**) The data matrix was substituted by residuals of the linear regression to subtract effects of age, gender, interaction age–gender, and the global effect (global mean cortical thickness) represented in (**c**). (**d**) Correlation matrix representing the concurrent changes among all pairs of anatomical structures. (**e**) The thresholding process for different sparsity levels to generate binary graphs. (**f**) Assessment of the network properties for all binary graphs obtained in (**e**).

The connectivity matrices obtained from bootstrapping were thresholded to create sparse binary graphs. We explored the Network Properties of the graphs over a range of sparsity degrees varied from 0.5 to 0.9 in steps of 0.02^41^. This range of sparsity degree has been indicated in previous studies to be optimal^42,43^. Similarly to other papers, only the positive correlation values are used to define the connectivity matrices. This choice is based on the lack of a clear physiological justification for negative correlations and the possible contamination by spurious negative correlation as a side effect of regressing out global effects in the preprocessing step (Fig. 2).

### Network properties analysis, graph theory approach

In general, a complex network can be represented as a graph G = [N, K], the components of this system are called nodes (N), and the relations or connections between them are called edges (K)^44^. The nodes are the anatomical regions, and the edges are the correlations in cortical thickness across subjects between pairs of these brain regions. It is important to note here that this is a mathematically derived network whose connections do not necessarily constitute brain functional or physiological mechanisms directly. However, these networks are based on structural data. Therefore, they indirectly reflect the underlying mechanism, allowing us at the same time to use them and their properties as possible biomarkers of the differences between normal and pathological brain states.

In particular, we analyzed the following global network attributes: clustering index^45^, characteristic path length^44,45^, local and global efficiency^46^, global connectivity, and homologous regions connectivity^47^. To describe the network's nodal properties, we computed the normalized betweenness centrality (NBC)^19,23^ measure to identify the network hubs. We also performed a modularity analysis representing a network with densely interconnected nodes and relatively few connections between nodes in different modules^48^. It is a reflection of the natural segregation within a network^49–51^. Additionally, we carried out a 'Targeted-Attack' study to evaluate the cortical thickness covariance network's resilience when the most critical regions (hubs) are virtually attacked. Definitions for these measures within the traditional interpretation of complex networks framework^52^ can be found in Supplementary Information and Table S2 online.

### Methodology for studying differences in regional cortical thickness

Cortical vertex-wise regression analyses were performed using the SurfStat MATLAB toolbox (http://www.math.mcgill.ca/keith/surfstat). Age, gender, and mean cortical thickness (CT) were statistically controlled. The statistical significance of the t-statistic maps for cortical thickness differences was corrected for multiple comparisons using Random Field Theory (RFT) to avoid false positives when more than 80,000 tests were performed^53^. RFT identifies statistically significant "clusters" of vertices and vertex "peaks". Cluster p-values show regions of connected vertices with p-values below 0.001 in clusters whose extent is significant at p < 0.05, i.e., a collection of connected vertices with p < 0.001 that was unlikely to occur by chance.

### Statistical methods to study ApoE4 modulation of global network properties

Network properties (NP) of the cortical thickness correlation matrices were computed for each sparsity degree values and different bootstrap samples in each MCI group. Thus, we had a set of Nboot = 2000 NP curves for each network property. The area under the curve (AUC) was computed for each network attribute to contrast the global behavior of these attributes^54^. The NP curves' monotonic changes make AUC a suitable descriptor of the networks' global performance.

We followed three main steps to examine differences in global network properties between groups: (1) construction of the empirical bootstrapped distribution of differences by subtracting the corresponding bootstrap samples between groups; (2) definition of the statistical significance level: a 95 percent confidence interval (CI) (biased corrected percentile bootstrap CI)^55^ of the distribution of the empirical difference is estimated; (3) Hypothesis testing: a significant difference between groups is accepted if CI does not contain zero, no significant difference is considered otherwise. A p-value associated with each hypothesis test is also reported.

### Methodology to explore nodal betweenness centrality (NBC) differences between groups

For each bootstrap sample of the cortical thickness connectivity matrix, the NBC was computed at every single sparsity degree. Previously to this process, the largest component^54^ of all bootstrap samples of the cortical thickness covariation matrices were calculated. The minimum sparsity degree for the largest connected components (equal to the number of structures) was used as an upper limit of the sparsity degree range. This step guarantees that all nodal NBCs come from fully connected cortical thickness networks. Similarly to global network properties, we take the AUC and follow the three main steps to examine differences between groups for each anatomical structure. To control for multiple comparisons (across the number of structures), we applied the False Discovery Rate (FDR) correction.

Hubs were selected as those with mean NBC superior to 1.5, similar to^41^.

Construction of the Cortical Thickness Network and computation of network metrics were performed using the MorphoConnect toolbox^56^ and subroutines of the Brain Connectivity Toolbox^52^ (https://sites.google.com/site/bctnet/). The figures were created using the BrainNet Viewer package^57^ (http://www.nitrc.org/projects/bnv) and the Gephi package^58^ (https://gephi.org/).

## Results

### Demographic and neuropsychological variables

There were no significant differences in gender, education, MMSE scores, and mean cortical thickness between groups (Table 1). However, the age was significantly different between MCI Carriers compared to non-Carriers (U (6542) = − 2.51, p = 0.01). The MCI Carriers group was younger than non-Carriers on the diagnosis age (74.17 vs. 76). This result agrees with previous studies were ApoE4 had been associated with a younger age of onset^59^.

**Table 1:** Demographics and neuropsychological variables for MCI groups.

	MCI		Statistics
	Carriers	Non-carriers	
N	126	127	–
Gender (M/F)	80/46	88/39	p = 0.33^&^
Age (y)	74.17 (6.91)	76 (7.97)	U(6542) = − 2.51, p = 0.01^+^
Age range	56.8–88.9	54.6–89.8	–
Education (y)	15.63 (3.03)	15.61 (3.38)	U(7878) = − 2.21, p = 0.83^+^
MMSE	26.97 (1.85)	27.17 (1.83)	U(7492) = − 0.87, p = 0.38^+^
Mean cortical thickness (mm)	2.23 (0.12)	2.23 (0.14)	U(7902) = − 0.17, p = 0.86^+^

Age, Education, MMSE, and Mean cortical thickness values are represented by means and standard deviations. Gender (M/F) is represented by the number of subjects. Significant set at p < 0.05. The superscripts “^&^” represents χ^2^ test; “^+^” represents the Mann–Whitney U test.

Key: *MCI* mild cognitive impairment; Carriers: *ApoE4*-positive; non-Carriers: *ApoE4*-negative; *M* male; *F* female; *y* years; *MMSE* mini-mental state examination.

### ApoE4-related changes in regional cortical thickness

Differences in cortical thickness between MCI Carriers and non-Carriers were not statistically significant after FDR correction (Supplementary Fig. S1 online). However, percent difference maps show trends for a reduced thickness bilaterally in the anterior temporal lobe and frontal lobe regions in the Carriers group compared with non-Carriers. The non-Carriers group tended to lower cortical thickness values in left posterior parietal areas, the precuneus, posterior cingulate gyrus, and frontal pole. For a list of clusters, see Supplementary Table S3 online.

### ApoE4 modulates the global network properties

**Figure 3:**
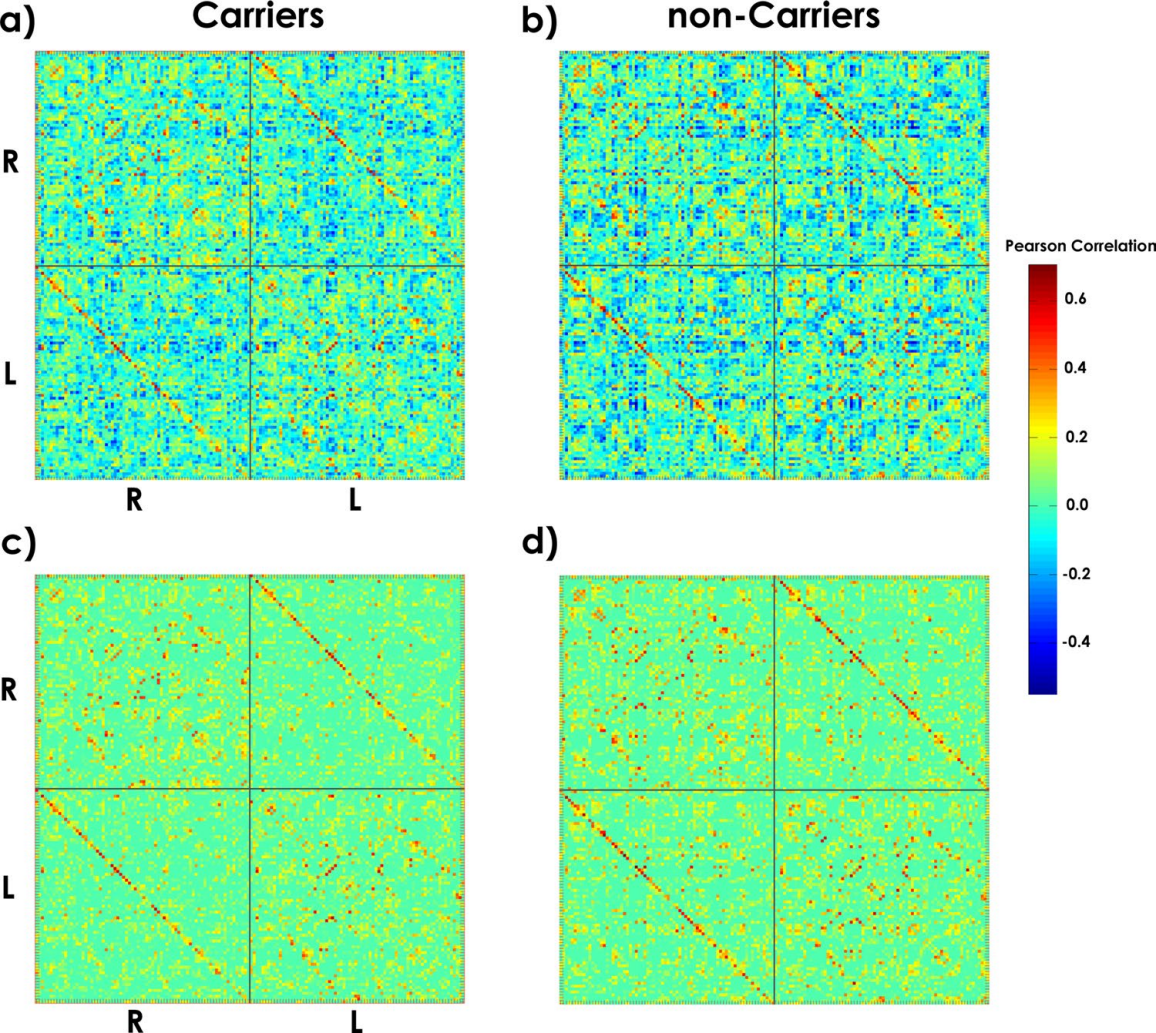
Cortical thickness correlation matrices for each group. (**a**) and (**b**) display matrices with positive and negative correlation values. (**c**) and (**d**) represent matrices with positive correlation values. The strength of the connection is indicated by the color bar. The 'R-'R' and 'L-'L' quadrants represent the intra-hemispheric cortical thickness correlations in the right and left hemispheres. The 'R-'L' and 'L-'R' quadrants depict the inter-hemispheric interactions. The diagonal of the 'L'-'R' quadrant shows the correlations in cortical thickness between homologous structures across hemispheres.

Figure 3 shows the cortical thickness matrices for negative (Fig. 3a,b) and positive correlation values (Fig. 3c,d) for each group. Only matrices with positive values were used for the subsequent analysis.

**Figure 4:**
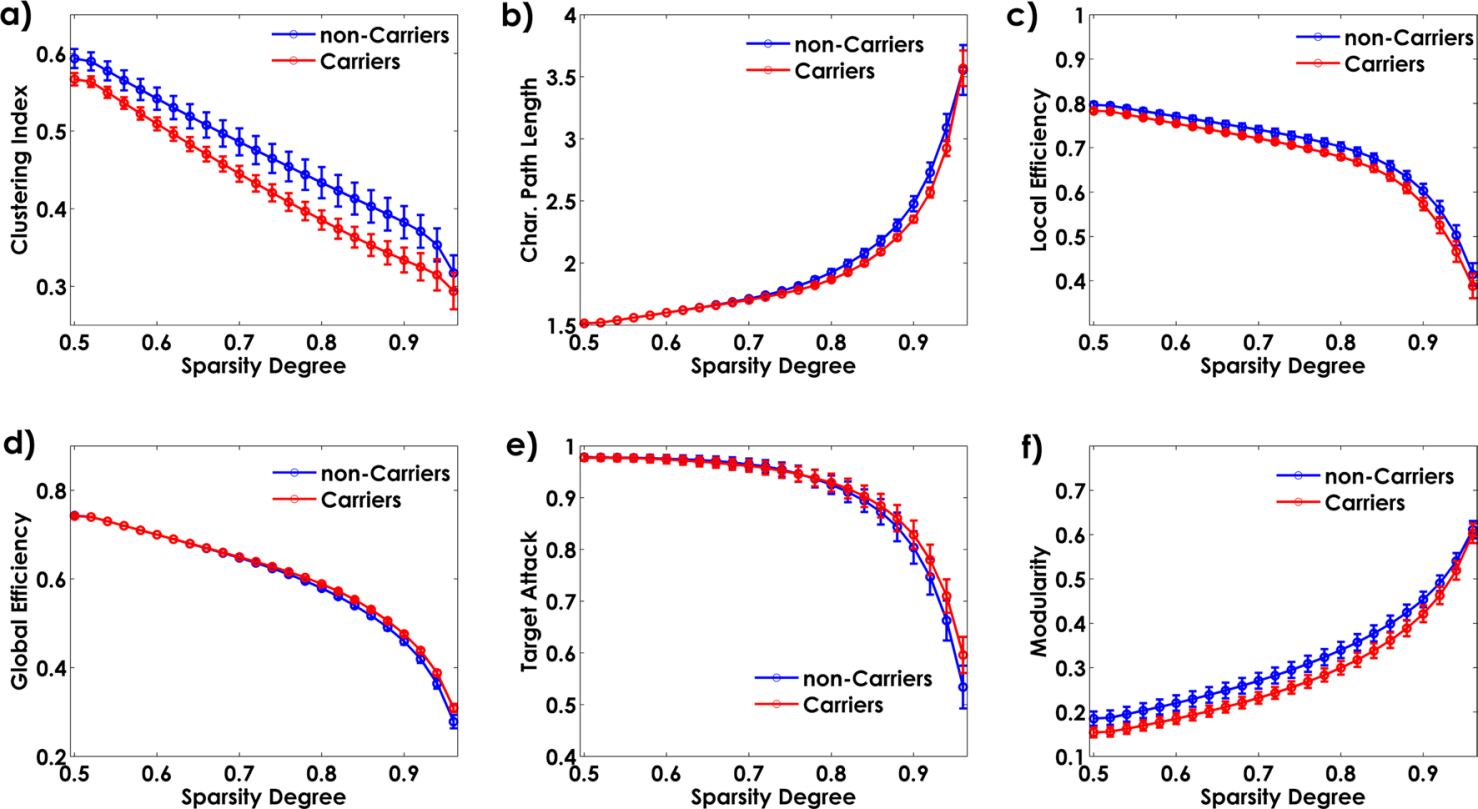
Global network properties as a function of sparsity degrees.

Figure 4 shows the changes in global network properties for both groups across various densities thresholds (0.5 to 0.9). The two networks exhibit differences in clustering index, characteristic path length, local and global efficiency, global connectivity, homologous regions connectivity, and modularity. The Target Attack simulation was not significantly different between groups (p > 0.05) for the whole range of densities values (Table 2).

**Table 2:** Network properties differences between groups.

Network property	Carriers	Non-carriers	Confidence interval (95%)	p-value
Clustering index	7.92 (0.2)	8.57 (0.3)	[− 1.62, − 0.61]	0.04*10^**–4**^
Characteristic path length	28.74 (0.06)	29.04 (0.15)	[− 0.88, − 0.28]	0.03*10^**–3**^
Target attack	16.24 (0.09)	16.25 (0.09)	[− 0.12, 0.42]	0.67
Local efficiency	12.39 (0.08)	12.70 (0.13)	[− 0.78, − 0.32]	0.09*10^**–4**^
Global efficiency	11.24 (0.01)	11.19 (0.03)	[0.04, 0.14]	0.06*10^**–3**^
Global connectivity	0.06 (0.01)	0.07 (0.01)	[− 0.01, − 0.001]	**0.04**
Homologous regions connectivity	0.36 (0.01)	0.4 (0.01)	[− 0.08, − 0.01]	**0.02**
Modularity	3.83 (0.20)	4.45 (0.28)	[− 1.61, − 0.29]	**0.02**

Network properties in each group are represented by the mean and standard deviations: mean (s.d). In bold, the significance differences, those confidence intervals that do not contain zero.

**Figure 5:**
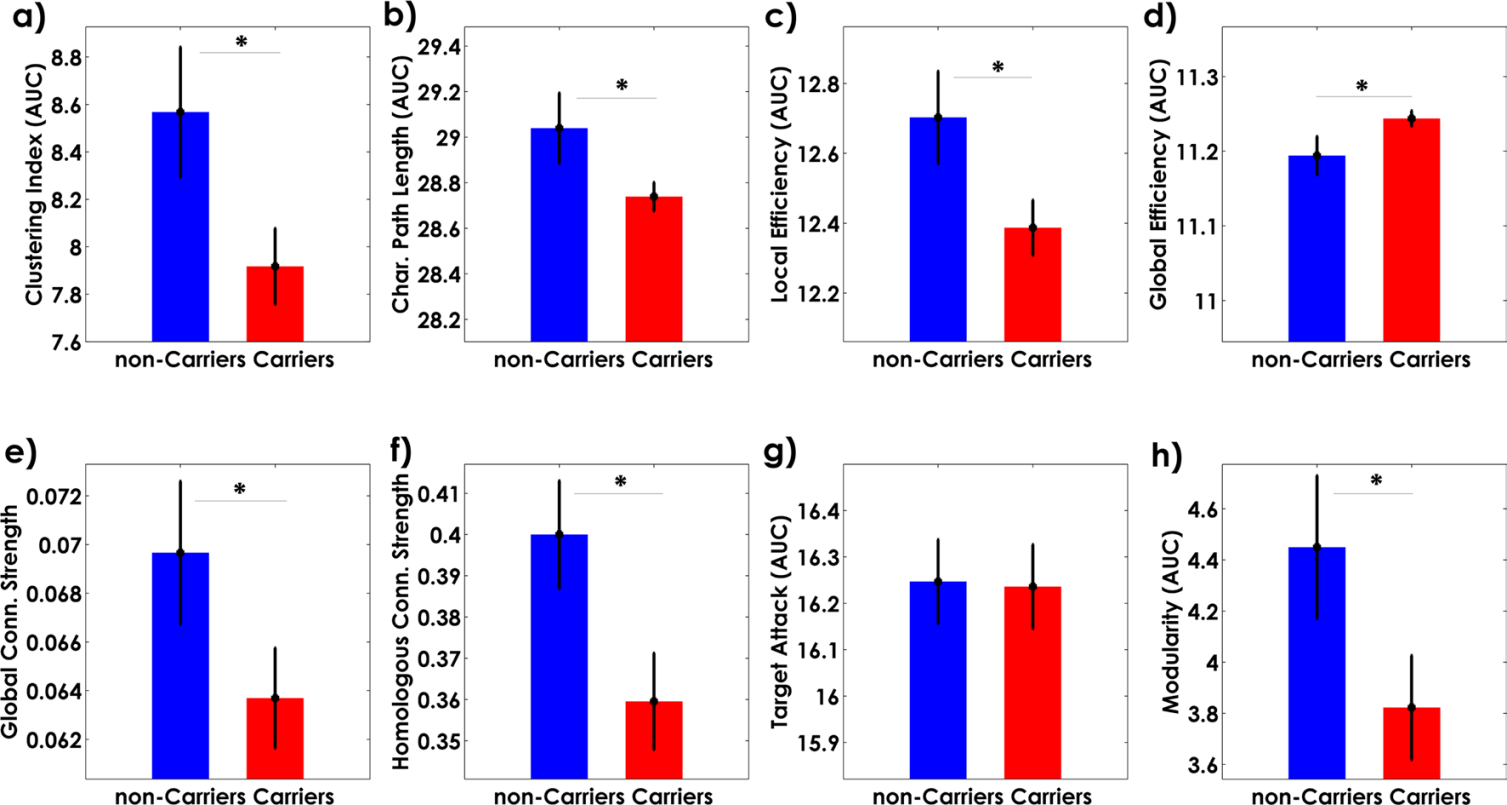
The area under the curves (AUC) of the cortical thickness covariance global properties. The bar heights represent the mean of the network properties, and the error bars are their standard deviation.

The comparison of the global network properties based on the AUC values revealed in MCI Carriers as compared with non-Carriers a decrease in clustering index, characteristic path length, local efficiency, homologous regions connectivity, global connectivity strength, and modularity. In contrast, the MCI Carriers group exhibited higher global efficiency (Fig. 5). The results of the statistical analysis, including confidence intervals, can be found in Table 2.

### Group-based differences in normalized betweenness centrality (NBC)

**Figure 6:**
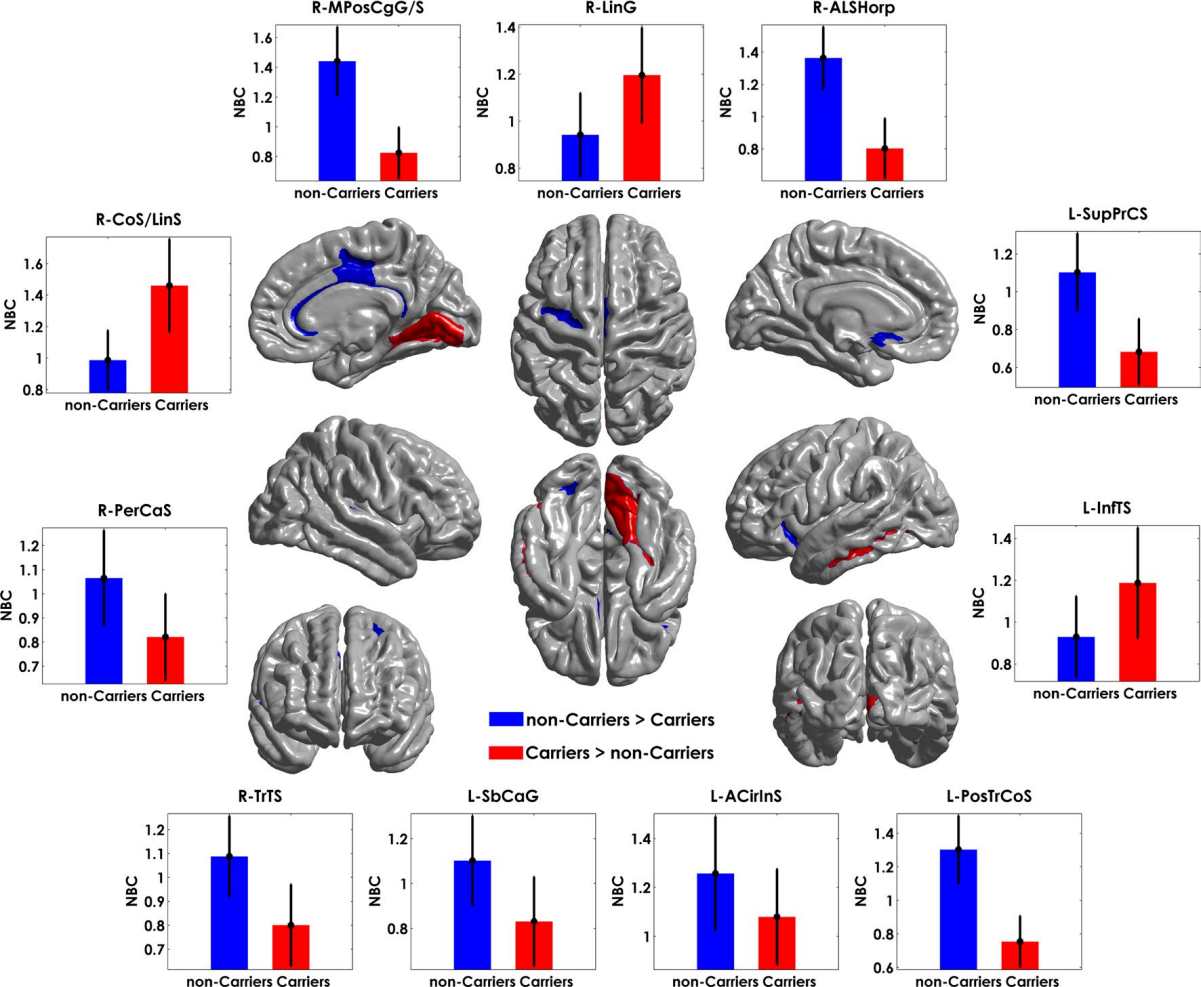
Significant differences between groups based on NBC. The bar heights represent the mean NBC values for each group, and the error bars the standard deviations. R: right hemisphere, L: left hemisphere. The regions were mapped onto the cortical surfaces using the BrainNet Viewer package (http://www.nitrc.org/projects/bnv).

We also studied the effects of ApoE4 on the Normalized Betweenness Centrality (NBC), a regional network property. The AUC analysis showed 11 regions with NBC differences between groups after FDR correction (p < 0.05) (Fig. 6). The full list of structures and the statistical analysis results (including confidence intervals) can be found in Supplementary Table S4 online. NBC regional differences between groups comprise mainly occipital-temporal brain areas followed by limbic and frontal regions. Compared with non-Carriers, Carriers showed lower NBC for all brain regions except for the right lingual gyrus, left inferior temporal sulcus, right medial occipitotemporal sulcus (collateral sulcus), and lingual sulcus.

### ApoE4 modifies the brain network hubs

**Figure 7:**
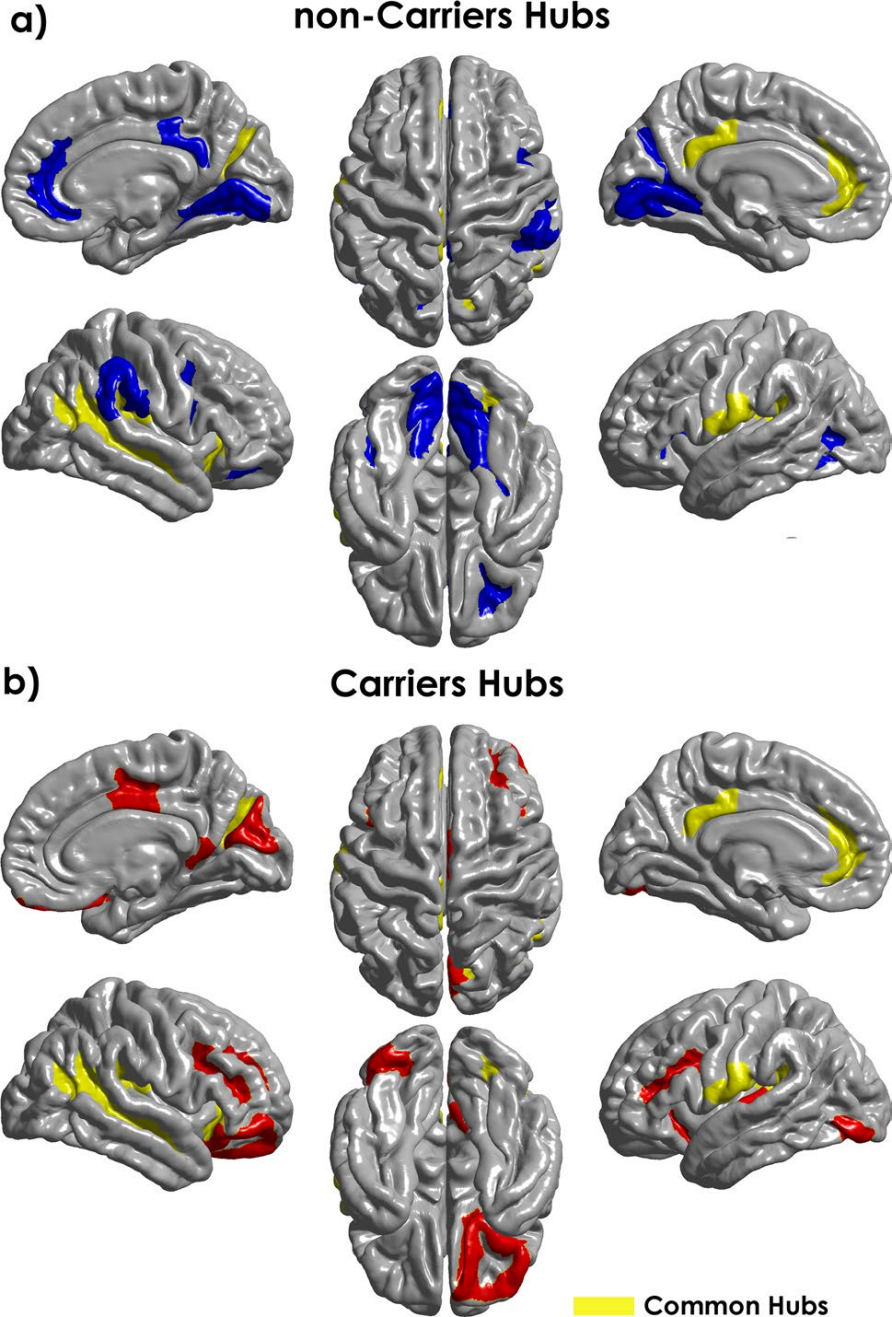
Hubs regions in MCI Carriers and non-Carriers based on the Normalized Betweenness Centrality (NBC). The blue regions represent hubs in the Carriers and the red ones the non-Carriers. In yellow are represent hubs common to both groups. The NBC values were mapped onto the cortical surfaces using the BrainNet Viewer package (http://www.nitrc.org/projects/bnv).

We also studied the effects on the hubs of the cortical thickness covariance network due to the presence of ApoE4. There were identified 24 hubs in each group (Fig. 7). The detailed list of structures and its NBC values were tabulated in Supplementary Table S5 online. We identify 11 common hubs to both groups (Fig. 7, yellow structures), including limbic (bilateral anterior part of the cingulate gyrus and sulcus, left posterior-dorsal part of the cingulate gyrus), insular (bilateral posterior ramus of the lateral sulcus, anterior segment of the circular sulcus of the insula), frontal (central operculum and sulci), temporal (superior temporal sulcus) and temporal-occipital and parietal-occipital regions (parieto-occipital sulcus, anterior transverse collateral sulcus).

Compared to non-Carriers, where hubs comprised mainly parietal-occipital-temporal areas, in the Carriers group were localized predominantly in frontal and parietal-occipital-temporal regions. Hub regions found only in Carriers, including the posterior-dorsal part of the cingulate gyrus, inferior occipital gyrus and sulcus, superior temporal and orbital gyrus. Areas identified as hubs in the non-Carriers comprised the lingual aspect of the medial occipitotemporal gyrus, supramarginal gyrus, and subcallosal gyrus.

### ApoE4-related change of the cortical thickness network modularity

**Figure 8:**
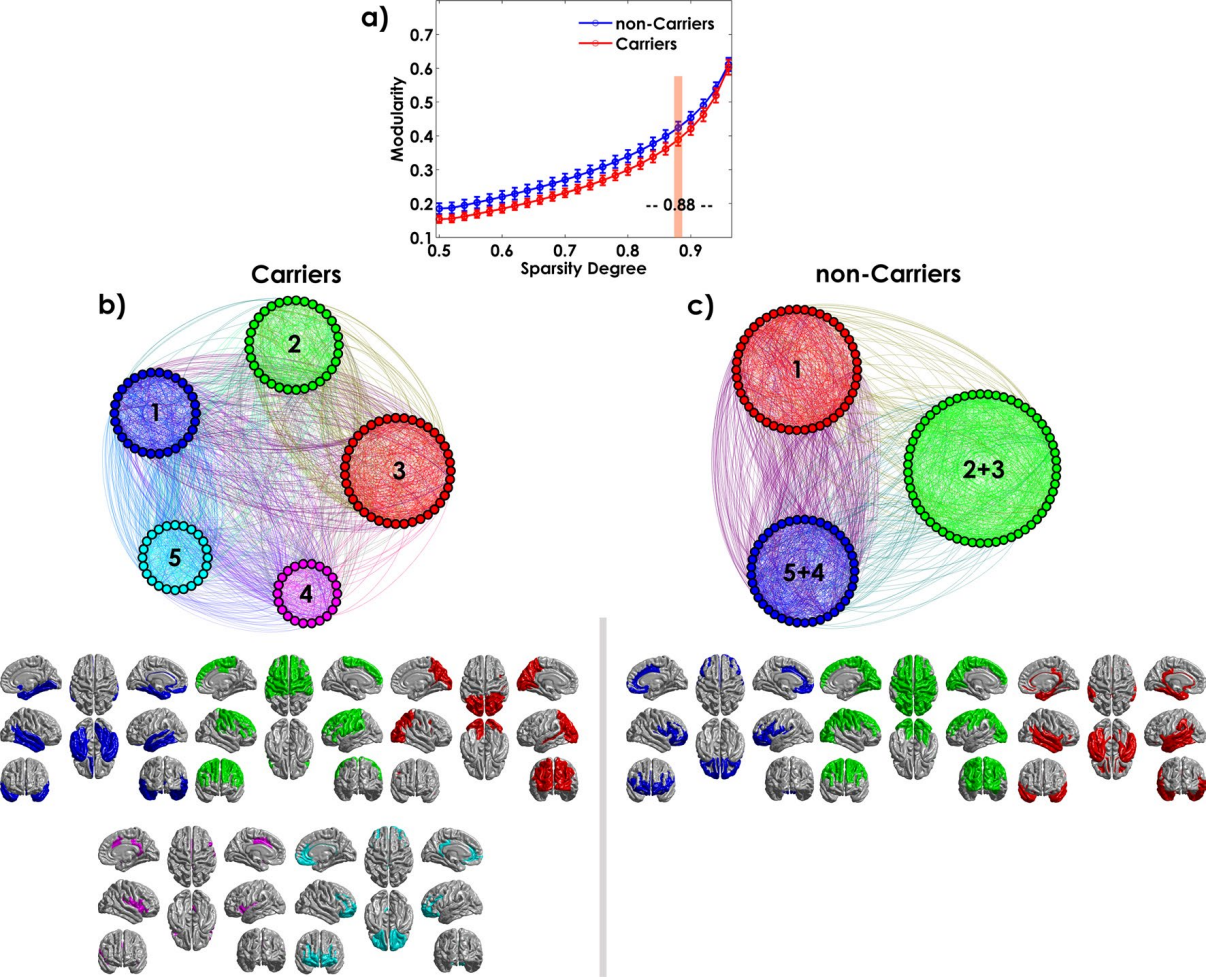
Module distributions for both Carriers and non-Carriers groups estimated using Newman's spectral community detection algorithm at sparsity degree 0.88. The circular representation of the modules was based on the Gephi package (https://gephi.org/). Inferior panels show the cortical surface mapping of the modules in both Carriers and non-Carriers groups using the BrainNet Viewer package (http://www.nitrc.org/projects/bnv). Each color represents those regions that belong to a specific module.

Modularity estimation was performed on the groups averaged connectivity matrix using Newman's metric. The resulting analysis (Fig. 8) divided the 148 cortical nodes into five modules for MCI Carriers and three modules in the non-Carriers group.

The modules are represented on a circular graph layout, where the nodes are placed in circles if they belong to the same module. Three communities defined the non-Carriers modularity with similar region numbers (community 1: N = 41, community 2: N = 59, and community 3: N = 48). Each module included a distributed set of regions. However, the analysis per lobule showed an anterior community based mainly in the frontal areas, a central module with similar frontal, parietal and occipital regions, and a posterior module integrated by temporal and temporal-occipital areas. On the other hand, Carriers showed more segregated modularity based on 5 communities with two large modules (community 2: N = 33 and community 3: N = 39) and three smaller ones (community 1: N = 30, community 4: N = 22, and community 5: N = 24). In this group, the community composition was more diverse as compared with non-Carriers. However, we were able to identify that the modules showed a specific pattern of regions summarized as follows: community (1) temporal-occipital, community (2) frontoparietal, community (3) parietal-occipital, community (4) insula, community (5) frontal regions. For a complete list of regions per module in each group, see Supplementary Table S6 online.

It is worth noting that modules 2 and 3 in Carriers are assigned to the second module in non-Carriers and modules 4 and 5 to module 3, suggesting a low level of segregation for non-Carriers. The sub-modules combine to form predominantly anterior–posterior large communities.

## Discussion

We investigated for the first time the cortical thickness structural covariance networks in ApoE4 Carriers and non-Carriers groups to assess the effect of genetic risk on large-scale network topology in MCI. Few previous studies have found evidence of the ApoE4 modulation on the MCI brain network topology using graph theory based on physiological variables derived from other image modalities (rs-fMRI, FDG-PET, and DWI). However, our approach is timely based on the following points:

1. As a morphometric descriptor, cortical thickness offers unique information about morphological covariance patterns between brain regions compared to other cortical measures^41^.
2. Structural covariance analysis is attractive because of the wide availability (in clinical and research settings) of high-quality sMRI scans compared with other modalities. Additionally, the cortical thickness derived from sMRI has proved consistent across scanner systems and field strengths^60^.
3. It has been shown that anatomical covariance patterns are related to functional and anatomical connectivity. However, comparing these connectivity measures has demonstrated that brain structural covariance networks capture complementary information of the same physiological processes^16,61^.
4. Graph theory provides a unique description of the multivariate neural process by looking at their local and global connectivity topology.
5. Unlike the previous studies^29–31^, we introduced the modularity and target attack analyses providing further information about the topological organization of the structural covariance networks in MCI.

Summarizing, our research presents novel experimental evidence regarding the ApoE4 effects on the brain network topology, which are worth investigating to define intermediate phenotypes in MCI.

In general, our findings revealed a decrease in global and homologous connectivity strength, clustering index, characteristic path length, local efficiency, modularity, and an increase of global efficiency in MCI Carriers compared to non-Carriers. MCI carriers showed lower values of NBC in several brain regions. Together, these findings concur with the evidence that ApoE4 is associated with an aberrant brain network topology in MCI. On the other hand, the changes are not detectable with the standard univariate approach based on the cortical thickness's differences. Our results support the concept that multivariate measures (i.e., covariations) combined with a graph theoretical approach are more sensitive to identifying complex pathological processes, as found in other brain diseases. Univariate measures derived from the standard methods could be insufficient for capturing subtle (early) abnormal changes.

Some of these results deserve more attention and will be discussed in the following.

In particular, we observed a decrease in the clustering coefficient index in MCI Carriers relative to non-Carriers, indicating lower local cortical thickness correlations. This finding suggests a topological organization more like a random network in this group of patients, a structure previously reported in AD subjects^18,62,63^. Moreover, it has been demonstrated an association between longitudinal decreases of the clustering index and risk of MCI conversion into AD^63^. Previous studies showed no differences between groups or reported similar results^29,31^. The disagreement between investigations could be related to several factors like group composition, sample size, different neuroimaging modalities, and atlas parcellation.

In the current study, we also observed a shorter characteristic path length associated with the presence of the ApoE4, indicating that fewer steps are required to carry on the information across remote brain regions^64^. A similar result was found in previous research in cognitively normal elderly ApoE4 Carriers^26^ using FDG-PET. A compensatory mechanism for early local pathological events seems a plausible hypothesis when the clustering index decreases in the presence of shorter characteristic path length. Also, the ApoE4 allele has been proposed as an example of antagonistic pleiotropy^65^. The concept means that ApoE4 may offer benefits during early and middle age and promote better compensatory mechanisms during early disease phases like MCI that can be captured using a complex network approach.

Like in the clustering index, one previous study reported no differences between groups in characteristic path length^31^. However, our results seem to be more reliable since Yao et al. (2015) gathered the MCI, AD, and healthy controls to form the Carriers and non-Carriers groups, making it challenging to disentangle group differences^31^.

Our analysis also showed a decrease in global connectivity strength- an aggregate measure of the correlation values between all possible pairwise anatomical structures- in MCI Carriers relative to non-Carriers. Previous studies did not report on this network property^29–31^. This finding may indicate that mechanisms underlying cortical thickness are differentially coordinated across this group of patients. Another possibility is that ApoE4 increases the interindividual differences between regional cortical thicknesses in Carriers. It may be due to less cortical thickness coordinated patterns concerning the homogeneity effects created by putative compensatory and shared brain region vulnerability associated with the aging processes and interactions with the MCI stage.

The nodal properties results allowed us to generate hypotheses about the ApoE4 impact on brain network integration and segregation in MCI. Similar to previous studies, we found the opposite effects of the ApoE4 genotype on nodal centrality^30,31^. There is probably more than one cause for these alterations, which makes disease-related changes in structural covariance challenging to interpret. A regional lower NBC may be suggestive of dysconnectivity due to a localized degeneration. By contrast, an increase may indicate over connectivity or synchronized cortical thickness loss in several areas targeted by the same neurodegenerative process. Most brain regions reported here are different from previous studies^30,31^ based on other neurophysiological variables. It is indicative that the networks of cortical thickness covariance capture supplementary information of the anatomical brain organization. Other factors could also be playing a role like sample characteristics and different statistical approaches.

Our findings showed agreements with previous studies in AD neurodegeneration. The fact that crucial structures like Posterior Cingulate Cortex (PCC) showed lower NBC values in the Carriers group suggests that regional topological properties may capture disease-related effects that can be further explored in association with the risk of AD progression. We identified between-group differences in NBC across different lobes, consistent with previous findings^26,30^. Several regions in the Limbic and Frontal cortex decreased centrality in Carriers as compared with non-Carriers. Notably, for all detected hubs, NBC was lower in Carriers than non-Carriers. Some of these structures were: cingulate gyrus, middle occipital gyrus, occipital pole, superior frontal sulci, and orbital gyrus. It is important to note that several of these regions are part of the Default Mode Network (DMN). As is recognized, the DMN is involved in self-referential functions such as episodic memory^66^ affected by AD. In this network, the PCC is a key integration node between the medial temporal lobe and medial prefrontal subsystems^66,67^. Previous studies reported in the DMN (including PCC) high glycolytic metabolism, enhancing abnormal amyloid deposition aggregation^68,69^. ApoE4, as a disrupted metabolic factor^10^, may alter the DMN resting-state activity and ultimately bringing atrophy in MCI ApoE4 Carriers, accelerating AD pathology early during the disease course.

Lower NBC values were also found in regions that belong to the Saliente Network (i.e.insula). This network operates on identified salience and, as such, includes known sites for sustained attention and working memory (dorsolateral prefrontal cortex, lateral parietal cortex), response selection (dorsomedial frontal), and response suppression (prefrontal cortex)^70–72^. Our findings may suggest that ApoE4 Carriers have altered regulation of control processes that subsequently influence memory performance.

To the best of our knowledge, we reported, for the first time, a modularity analysis of the structural covariance network in MCI. We observed a decreased modularity in Carriers as compared with non-Carriers. A less modular network implies fewer connections within modules and more connections to other modules. In graph theory terms, Carriers shows better cost-efficiency wiring regarding the physical volume occupied, conduction delay, and metabolic cost. On the other hand, the increase of interconnectedness between modules can lead to the rapid spreading of disease pathological markers (neurodegenerative process) and loss of specialization^73^. In many networks, as in our case, modularity and global efficiency are inversely related, as a highly modular topology could require long communication paths to integrate information across the network.

In addition to these differences, the module size and composition also change associated with the ApoE4. This analysis revealed in Carriers a spatial rearrangement of these communities. They include sets of brain regions that are anatomically proximal, and they mainly belong to the same lobule. However, in non-Carriers, the module's compositions are more distributed across the cortex. We identified anterior-medial-posterior network modularity mainly formed by frontal, frontal-parietal, and temporal-occipital modules. This modular topology has been described previously in resting-state networks in normal aging. It evidences the brain network evolves from a preferentially local connectivity pattern to a more distant and functionally community structure^74^. Further longitudinal studies on modularity patterns differences between Carriers and non-Carriers could offer an exciting opportunity to distinguish those MCI patients at high risk of AD progression.

In conclusion, our study applies the graph theory to assess the ApoE4-related changes on global and local network topology in MCI based on the concurrent variations of the cortical thickness across anatomical structures. Our findings showed that some network properties changes in MCI Carriers seem to be associated with altered communication between neighboring regions. It may be an early response to AD-related pathological markers (i.e., tau-tangles and amyloid-beta plaques depositions). On the other hand, a better global network communication could be considered the expression of compensatory/degeneracy mechanisms to sustain the transmission of the information across distant brain regions associated with the genetic challenge. These changes in topological attributes may be considered sensitive markers to detect early brain network changes related to the disease progression.

The methodological approach used in this study has several limitations. The structural covariance analysis has a static nature. Evidence suggests that the brain undergoes spontaneous reconfiguration at a temporal scale^75^, as such properties like modular structure and hub may fluctuate over time. A quantitative comparison of the network topological attributes between studies is difficult. These properties' values depend on experimental parameters like brain parcellation, nodes-edge definitions, and sample size^76^. Despite providing useful information, structural covariance analysis is a group approach. It does not allow individual analysis and statistical associations between topological network attributes and clinical/cognitive measures.

Other aspects need to be addressed in future investigations. (1) MCI patients exhibit different progression trajectories that we did not consider here; accordingly, further follow-up longitudinal studies are warranted to examine the interaction between network properties, disease progression, and ApoE4 (2) The inclusion of healthy elderly sample, as well as AD patients, would help to fully characterize the ApoE4 effect on the brain network properties across the disease spectrum (4) This study did not investigate whether the ApoE4-related impact on the brain network topology is mediated by pathological disease markers like beta-amyloid and tau deposition. Further investigations on this topic will clarify the underlying mechanisms associated with the brain network properties changes. Despite these limitations, our study sheds light on the structural connectomics of MCI associated with the ApoE4. We considered a complex network analysis with the genetic risk factors inclusion, a valuable approach to understanding the AD spectrum, which could improve the personalized medicine perspective.

## Supplementary Information

Supplementary Information.

## Data Availability

Data used in the preparation of this article were obtained from the Alzheimer's Disease Neuroimaging Initiative (ADNI) database (adni.loni.usc.edu). ADNI database is publicly accessible from adni.loni.usc.edu upon request. ADNI's primary goal has been to test whether serial MRI, PET, other biological markers, and clinical and neuropsychological assessment can be combined to measure the progression of mild cognitive impairment and early Alzheimer's Disease. The Principal Investigator of ADNI is Michael W. Weiner, MD (email: Michael.Weiner@ucsf.edu).
