# Supplementary material for: Apolipoprotein E4 effects on topological brain network organization in mild cognitive impairment
Source: Sci Rep. 2021 Jan 12;11:845. doi: 10.1038/s41598-020-80909-7 (PMC7804004; doi:10.1038/s41598-020-80909-7)
Supplement: Supplementary file 1 — Supplementary Information. [file 41598_2020_80909_MOESM1_ESM.pdf]

Title: Apolipoprotein E4 effects on Topological Brain Network Organization in Mild Cognitive Impairment

Authors: Gretel Sanabria-Diaz, Lester Melie-Garcia, Bogdan Draganski, Jean-Francois Demonet, Ferath Kherif

Supplementary Information

Supplementary Table S1. List of anatomical structures

Code	Atlas Name	Structure Full name	Structure Short Name	Lobe
1	G and S frontomargin	Fronto-marginal gyrus (of Wernicke) and sulcus	FMarG.S	F
2	G and S occipital inf	Inferior occipital gyrus (O3) and sulcus	InfOcG.S	T -O
3	G and S paracentral	Paracentral lobule and sulcus	PaCL.S	F-P
4	G and S subcentral	Subcentral gyrus (central operculum) and sulci	SbCG.S	F
5	G and S transv frontopo	Transverse frontopolar gyri and sulci	TrFPoG.S	F
6	G and S cingul Ant	Anterior part of the cingulate gyrus and sulcus (ACC)	ACgG.S	L
7	G and S cingul Mid Ant	Middle-anterior part of the cingulate gyrus and sulcus (aMCC)	MACgG.S	L
8	G and S cingul Mid Post	Middle-posterior part of the cingulate gyrus and sulcus (pMCC)	MPosCgG.S	L
9	G cingul Post dorsal	Posterior-dorsal part of the cingulate gyrus (dPCC)	PosDCgG	L
10	G cingul Post ventral	Posterior-ventral part of the cingulate gyrus (vPCC, isthmus of the cingulate gyrus)	PosVCgG	L
11	G cuneus	Cuneus (O6)	Cun	O
12	G front inf Opercular	Opercular part of the inferior frontal gyrus	InfFGOpp	F
13	G front inf Orbital	Orbital part of the inferior frontal gyrus	InfFGOrp	F
14	G front inf Triangul	Triangular part of the inferior frontal gyrus	InfFGTrip	F
15	G front middle	Middle frontal gyrus (F2)	MFG	F
16	G front sup	Superior frontal gyrus (F1)	SupFG	F
17	G Ins lg and S cent ins	Long insular gyrus and central sulcus of the insula	LoInG.CInS	I
18	G insular short	Short insular gyri	ShoInG	I
19	G occipital middle	Middle occipital gyrus (O2, lateral occipital gyrus)	MOcG	O
20	G occipital sup	Superior occipital gyrus (O1)	SupOcG	O
21	G oc temp lat fusifor	Lateral occipito-temporal gyrus (fusiform gyrus, O4-T4)	FuG	T-O
22	G oc temp med Lingual	Lingual gyrus, lingual part of the medial occipito-temporal gyrus, (O5)	LinG	O-T

23	G oc temp med Parahip	Parahippocampal gyrus, parahippocampal part of the medial occipito-temporal gyrus, (T5)	PaHipG	O-T
24	G orbital	Orbital gyri	OrG	F
25	G pariet inf Angular	Angular gyrus	AngG	P
26	G pariet inf Supramar	Supramarginal gyrus	SuMarG	P
27	G parietal sup	Superior parietal lobule (lateral part of P1)	SupPL	P
28	G postcentral	Postcentral gyrus	PosCG	P
29	G precentral	Precentral gyrus	PrCG	F
30	G precuneus	Precuneus (medial part of P1)	PrCun	P
31	G rectus	Straight gyrus, Gyrus rectus	RG	F
32	G subcallosal	Subcallosal area, subcallosal gyrus	SbCaG	L
33	G temp sup G T transv	Anterior transverse temporal gyrus (of Heschl)	HG	T
34	G temp sup Lateral	Lateral aspect of the superior temporal gyrus	SupTGLp	T
35	G temp sup Plan polar	Planum polare of the superior temporal gyrus	PoPI	T
36	G temp sup Plan tempo	Planum temporale or temporal plane of the superior temporal gyrus	TPI	T
37	G temporal inf	Inferior temporal gyrus (T3)	InfTG	O-T
38	G temporal middle	Middle temporal gyrus (T2)	MTG	T
39	Lat Fis ant Horizont	Horizontal ramus of the anterior segment of the lateral sulcus (or fissure)	ALSHorp	F
40	Lat Fis ant Vertical	Vertical ramus of the anterior segment of the lateral sulcus (or fissure)	ALSVerp	F
41	Lat Fis post	Posterior ramus (or segment) of the lateral sulcus (or fissure)	PosLS	I
42	Pole occipital	Occipital pole	OcPo	T-O
43	Pole temporal	Temporal pole	TPo	T-O
44	S calcarine	Calcarine sulcus	CcS	O
45	S central	Central sulcus (Rolando's fissure)	CS	F
46	S cingul Marginalis	Marginal branch (or part) of the cingulate sulcus	CgSMarp	F
47	S circular insula ant	Anterior segment of the circular sulcus of the insula	ACirInS	I
48	S circular insula inf	Inferior segment of the circular sulcus of the insula	InfCirInS	I
49	S circular insula sup	Superior segment of the circular sulcus of the insula	SupCirInS	I
50	S collat transv ant	Anterior transverse collateral sulcus	ATrCoS	O-T
51	S collat transv post	Posterior transverse collateral sulcus	PosTrCoS	O-T
52	S front inf	Inferior frontal sulcus	InfFS	F
53	S front middle	Middle frontal sulcus	MFS	F
54	S front sup	Superior frontal sulcus	SupFS	F
55	S interm prim Jensen	Sulcus intermedius primus (of Jensen)	JS	P
56	S intrapariet and P tra	Intraparietal sulcus (interparietal sulcus) and transverse parietal sulci	IntPS.TrPS	P
57	S oc middle and Lunatus	Middle occipital sulcus and lunatus sulcus	MOcS.LuS	O
58	S oc sup and transversa	Superior occipital sulcus and transverse occipital sulcus	SupOcS.TrOcS	O
59	S occipital ant	Anterior occipital sulcus and preoccipital notch (temporo-occipital incisure)	AOcS	T-O
60	S oc temp lat	Lateral occipito-temporal sulcus	LOcTS	O-T
61	S oc temp med and	Medial occipito-temporal sulcus (collateral	CoS.LinS	O-T

	Lingu	sulcus) and lingual sulcus		
62	S orbital lateral	Lateral orbital sulcus	LOrS	F
63	S orbital med olfact	Medial orbital sulcus (olfactory sulcus)	MedOrS	F
64	S orbital H Shaped	Orbital sulci (H-shaped sulci)	OrS	F
65	S parieto occipital	Parieto-occipital sulcus (or fissure)	POcS	P-O
66	S pericallosal	Pericallosal sulcus (S of corpus callosum)	PerCaS	L
67	S postcentral	Postcentral sulcus	PosCS	P
68	S precentral inf part	Inferior part of the precentral sulcus	InfPrCS	F
69	S precentral sup part	Superior part of the precentral sulcus	SupPrCS	F
70	S suborbital	Suborbital sulcus (sulcus rostrales, supraorbital sulcus)	SbOrS	F
71	S subparietal	Subparietal sulcus	SbPS	P
72	S temporal inf	Inferior temporal sulcus	InfTS	T-O
73	S temporal sup	Superior temporal sulcus (parallel sulcus)	SupTS	T
74	S temporal transverse	Transverse temporal sulcus	TrTS	T

The regions are listed following the anatomical brain atlas described in Destrieux et al. (2010). The subcortical regions were not included. The short names, as well as the full structure names, are specified. The lobe to which the structure belongs is also included. L: Limbic; I: Insula; P: Parietal; O: Occipital; F: Frontal; T: Temporal. G: gyri; S: sulcus.

Supplementary Table S2. Network properties definition and interpretation.

Network Property	Definition	Interpretation
**Measures of functional brain segregation**<br>*Functional segregation: the ability for specialized processing to occur within densely interconnected groups of brain regions*		
**Clustering index**	. Nodes are considered neighbors when a connection between them exists, which is not reduced to a physical neighborhood concept.	In anatomical networks, the clusters suggest the potential for functional segregation, while the presence of clusters in functional networks suggests an organization of statistical dependencies indicative of segregated neural processing.
**Modularity**	Many complex networks, like the brain, consisting of several modules. Modules are derived from a decomposition of the network into subcomponents that are internally strongly coupled but externally only weakly correlated.<br>Each module contains several densely interconnected nodes (brain regions).	Dense connectivity within modules allows brain regions within each module to interact with one another easily. In contrast, sparser connectivity between modules allows each set of brain regions to be relatively independent of one another (specialized functions).<br>Diminished connectivity between communities can result in loss of essential interactions or even disconnection of an entire community. On the other hand, excessive connectivity between modules may result in loss of compartmentalization or specialization of this brain region group.
	It is the average efficiency of the local	This measure reveals how much the

**Local Efficiency**	subgraphs	brain as a system is fault-tolerant, showing how efficient the communication is among the first neighbors of a node (brain region) when it is removed.
**Measures of functional brain integration**<br><br>*Functional integration: is the ability to combine specialized information from distributed brain regions rapidly*		
**Characteristic path length**	The path length is the minimum number of edges that must be traversed to go from one node (brain region) to another. It is a measure of the typical separation between two brain regions. The average shortest path length between all pairs of nodes in the the network is known as the characteristic path length of the network.<br>Connection lengths are typically dimensionless and do not represent spatial or metric distance.	Lengths of paths consequently estimate the potential for functional integration between brain regions. Shorter paths are implying a more substantial potential for integration between brain regions. Paths in functional/morphological networks represent statistical associations and may not correspond to information flow on anatomical connections. In this case, paths are less straightforward to interpret in terms of brain functions.
**Global efficiency**	It is the average inverse of the shortest path length.	The global efficiency es primarily affected by the shorth path length, represents a superior measure of integration.
**Global connectivity**	It summarizes the interregional correlations coefficients between all possible pairs of nodes (brain regions). Describes the degree to which nodes are connected in a network. It can be quantified based on network metrics such as the relative density, the shortest path, or the diameter of the network.	Previous studies have found strong correlations between regions with no direct structural (white matter tracts) connection. The total interregional morphometric correlations could capture all indirect structural correlations between two brain regions facilitated by a third party, from which diverse factors such as pathologic changes to the connectivity patterns could be detected.
**Normalized betweenness centrality**	The centrality of a node (brain region) measures how many of the shortest paths between all other brain regions pairs in the network pass through it. Bridging nodes that connect disparate parts of the network have a high betweenness centrality.	A node (brain region) with high centrality is thus crucial to efficient communication. It is based on the idea that central nodes participate in many short paths within a network, and consequently, act as essential controls of information flow.<br>Their loss is particularly disruptive to the brain network. Several regions in the frontal and parietal cortex have high centrality in the human brain, particularly the posterior cingulate and precuneus. These are areas of the brain defined as transmodal or heteromodal. They are involved in integrating processing across several cognitive modalities. Some of these regions overlap with the DMN, while others coincide with the frontoparietal system.
	Hubs are nodes with a high degree or	Hubs often interact with many other

**Hubs**	high centrality. Measures of node centrality assess the importance of individual nodes.<br>The hubs of the network are the regions with high values of NBC.	areas, facilitate functional integration, and play a vital role in the brain network resilience to insult. Hubs are a cost-efficient solution to increase network efficiency to support cognitive processes without requiring many metabolically expensive connections.<br>Hubs are suggested to be essential for cognition because they are located along the shortest paths in the network, and therefore are likely to play a critical role in distributed patterns of communication.<br>This location is evident both by their high degree and by their tendency to connect, forming a core or "rich-club" that boosts inter-hub communication's robustness and promotes efficient communication across the brain.<br>Damage to brain hubs is expected to have critical consequences for cognitive function in terms of the severity and pervasiveness of cognitive deficits.
**Measures of network resilience**<br>*Reflects the brain network vulnerability to insults*		
**Targeted Attack**	The importance of an individual node to network efficiency can be assessed by deleting it and estimating the the efficiency of the 'lesioned' network.<br>It is an indirect measure of resilience that reflects network vulnerability to insult. Complex networks like the brain are highly vulnerable to disruptions of the central node (hubs).	Robustness refers either to the network's structural integrity following the deletion of nodes or edges or to the effects of perturbations on local or global network states.<br>Direct measures of network resilience generally test the network before and after a presumed insult by computationally simulated targeted removal of nodes and links. The effects of such lesions on the brain network may then be quantified by characterizing changes in the resulting brain connectivity.

Sample Biomarkers Characteristics

Cerebrospinal fluid (CSF) samples at baseline were collected from 192 MCI subjects as part of the ADNI-1 protocol. The overlap between this sample and the one selected for the present study corresponds to 132 subjects (67 MCI-Carriers and 65 MCI non-Carriers). For details about CSF samples and methods see UPENN CSF Biomarkers Elecsys [ADNI1,GO,2].csv and UPENN CSF Biomarkers Elecsys METHODS [ADNI1,GO,2] (PDF) at https://ida.loni.usc.edu/pages/access/studyData

MCI groups characterization based on ADNI1 CSF biomarker measurements (Aβ (1-42), tTau, and pTau) at baseline

Biomarkers	**MCI-Carriers** (N,67)	**MCI non-Carriers** (N, 65)
**Aβ(1-42)***	710.88 (398.26)	1068.54 (528.09)
**tTau***	335.79 (125.94)	276.52 (109.45)
**pTau***	35.77 (16.90)	26.05 (11.93)

Values are represented by the mean (pg/mL) and the Standard deviations (SD). N: number of subjects; MCI: Mild Cognitive Impairment; Carriers: *ApoE4*-positive; non-Carriers: *ApoE4*-negative; Aβ(1-42): β-Amyloid (1-42); tTau: Total-Tau; pTau: Phospho-Tau. The superscript "*" represents significant T-test for independent samples set at $p<0.05$

MCI groups characterization as biomarker positive and negative based on Aβ (1-42), tTau and pTau

	**Group**			
	**MCI-Carriers**		**MCI non-Carriers**	
**Biomaker**	BM+	BM-	BM+	BM-
**Aβ(1-42)**	56 (83.9)	11(16.4)	30 (46.1)	35 (53.8)
pTau	45 (67.2)	22 (32.8)	24 (36.9)	41 (63)
tTau	41 (61.2)	26 (38.8)	22 (33.8)	43 (66.1)
***pTau/* Aβ(1-42)**	55 (82)	12 (17.9)	29 (44.6)	36 (55.2)
***tTau/* Aβ(1-42)**	56 (83.6)	11 (16.4)	28 (43)	37 (56.9)

Values are represented by the number of subjects (%). The positive (+) and negative (-) status are based on PET-optimized cut-offs for Aβ(1-42), pTau/Aβ(1-42) and tTau/Aβ(1-42). CSF, cerebrospinal fluid; BM, biomarker; Aβ(1-42): β-Amyloid (1-42); tTau: Total-Tau; pTau: Phospho-Tau; MCI, mild cognitive impairment. For biomarkers, cut-offs description, see Hansson et al. (2018).

CSF Biomarkers groups characterization. The Amyloid plaque burden +/- represented by Aβ (1-42) and Tau pathology represented by tTau+ or -

Biomaker	MCI-Carriers	MCI non-carriers
Aβ(1-42)+/ tTau +	37 (55.2%)	5 (7.5%)
Aβ(1-42)+/ tTau -	19 (28.4%)	25 (37.3%)
Aβ(1-42)-/ tTau +	4 (5.9%)	17 (25.6%)
Aβ(1-42)-/ tTau -	7(10.5%)	18 (26.9%)

Values are represented by the number of subjects (%). The positive (+) and negative (-) status are based on PET-optimized cut-offs for Aβ(1-42), pTau/Aβ(1-42) and tTau/Aβ(1-42). CSF, cerebrospinal fluid; Aβ(1-42): β-Amyloid

(1-42); tTau: Total-Tau; pTau: Phospho-Tau; MCI, mild cognitive impairment. For biomarkers, cut-offs description, see Hansson et al. (2018).

Graph Theory Metrics

The following group theoretical metrics were computed in the present study:

Clustering index (C). The clustering index of a node 'i' is defined as the number of existing connections between the node's neighbors divided by all possible connections. It is a measure of the inherent tendency to cluster nodes into strictly connected neighborhoods. Nodes are considered neighbors when a connection between them exists, which is not reduced to a physical neighborhood concept. The clustering index for the whole graph G is defined as the average clustering around each node:

$$C = \frac{1}{N}\sum_{i \in G} C_i \tag{1}$$

Represent the number of nodes. Clearly, 0 < C < 1; and C= 1 if and only if the network is fully connected, that is, each node is connected to all other nodes.

Characteristic path length (L). The characteristic path length L of the graph G is the smallest number of connections required to connect one node to another, averaged over all pairs of nodes. It is a measure of the typical separation between two nodes (structures) i and j $(\forall i, j \in N)$, and it is defined as the mean of geodesic lengths d_{ij} over all pairs of nodes.

$$L = \frac{1}{N(N-1)}\sum_{\substack{i,j \in G \\ i \neq j}} d_{ij} \tag{2}$$

In the unweighted network context, the geodesic length d_{ij} is defined as the number of edges along the shortest path connecting nodes i and j.

Nodal efficiency (E_{glob}, E_{loc}). The concept of efficiency has also been expressed in terms of information flow. That is, small-world networks are very efficient in terms of global and local communication and they are defined to have high global E_{glob} and local E_{loc} efficiency. The global E_{glob} of a graph G is expressed as:

$$E_{glob} = \frac{1}{N\left(N-1\right)} \sum_{\substack{i,j \in G \\ i \neq j}} \frac{1}{d_{ij}} \tag{3}$$

This measure reflects how efficiently the information can be exchanged over the network, considering a parallel system in which each node sends information concurrently through the network. On the other hand, the E_{loc} of G is defined as the average efficiency of the local subgraphs:

$$E_{loc} = \frac{1}{N} \sum_{i \in G} E_{glob}\left(G_i\right) \tag{4}$$

Where G_i is the subgraph of the neighbors of '*i*'. This measure reveals how much the system is fault-tolerant, showing how efficient the communication is among the first neighbors of *i* when it is removed. As above, nodes are considered neighbors when a connection between them exists, which is not reduced to a physical neighborhood concept.

Global and Homologous regional connectivity. We assessed the global connectivity and homologous region connectivity. First, the absolute correlation coefficient values were converted to z using Fisher's r-to-z transformation, followed by taking the mean and transforming back to correlations through the inverse Fisher's z-to-r transformation. All anatomical regions were used to estimate the global connectivity, whereas only the correlation values between homologous regions were used in the mean homologous region connectivity.

Nodal centrality: normalized betweenness centrality (NBC). The ‘betweenness centrality’ B_i of a node *i* is defined as the number of shortest paths between any two nodes that run through node *i*. We measured the normalized betweenness centrality as $b_i = B_i / \langle B \rangle$, where $\langle B \rangle$ was the average betweenness of the network. b_i is a global centrality measure that captures a node's influence over information flow between other nodes in the network. In our case, betweenness centrality b_i could be used to reflect the effects of ApoE4 on the global roles of regions in the cortical thickness covariance networks. Hubs were selected as those with b_i superior to 1.5, similar to previous investigations.

Modularity. A complex network module is a subset of nodes that are densely connected within the modules but sparsely connected between the modules. Here we have adopted Newman's metric as a modularity measure to compare our results with previous studies that used this method in other neuroimaging modalities.

Targeted Attack: Methodology to study the robustness of the cortical thickness covariance network

We calculated a surrogate measure of the resilience of the cortical thickness covariance network against a targeted attack. In a simulated targeted attack study, network hubs are removed one by one in order of betweenness centrality (NBC). Each time a node was removed from the network, the largest connected component's size was recomputed. We defined the robustness parameter as the AUC showing the relative largest connected component's size versus the number of nodes removed[94]. Robust networks retain large connected components even when several nodes have been knocked out, represented by a large AUC. As before, we repeated this procedure for all bootstrapped connectivity matrices and sparsity degrees. The same statistical procedure used for

evaluating the ApoE4 effect of global network properties was applied to explore network robustness differences between groups.

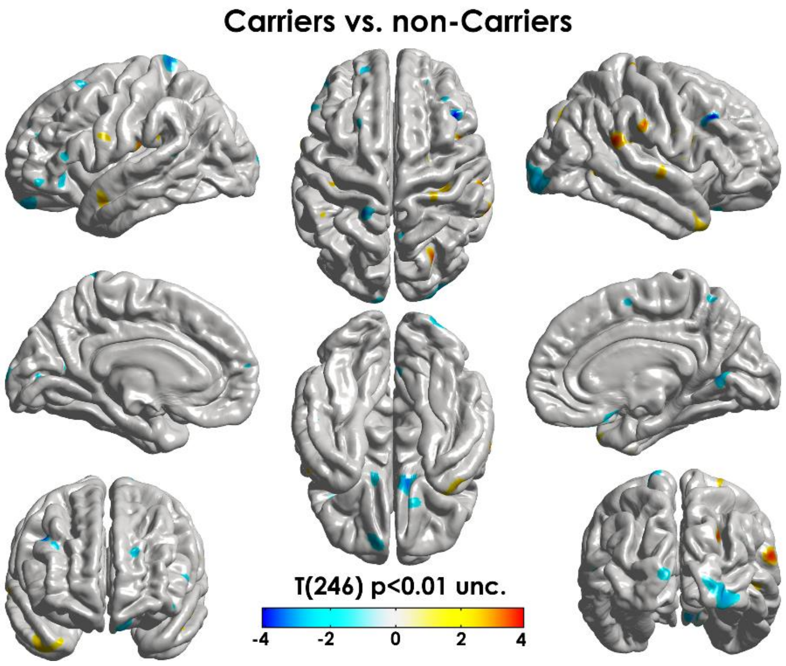

Fig S1. Spatially distributed differences in cortical thickness between MCI Carriers and non-Carriers (p<.01, uncorrected). Relative deficits in Carriers compared with non-Carriers are displayed in red/yellow, while excesses are shown in blue/cyan. Surfaces are presented in lateral, medial, and frontal views for the left and right pial (outer) surface. After Random-field theory-based cluster-corrected (q<.05) there were no clusters of significant differences between groups.

Supplementary Table S3. Cortical thickness cluster differences between MCI Carriers vs non-Carriers for p<0.01 (uncorrected)

Structure Name	t-Student	p-value
rh G front middle	-4.10	3E-05
rh S front inf	-4.00	4E-05
rh S precentral-inf-part	3.92	6E-05
rh G pariet inf-Supramar	3.75	1E-04
lh S oc-temp med and Lingual	3.69	1E-04
rh G occipital sup	3.61	2E-04
rh S orbital med-olfact	-3.40	4E-04
lh G and S paracentral	-3.39	4E-04
rh Medial wall	-3.37	4E-04
rh G rectus	-3.37	4E-04
lh G and S subcentral	3.35	5E-04
lh G pariet inf-Supramar	3.34	5E-04
lh G parietal sup	-3.26	6E-04

rh G oc-temp med-Lingual	-3.18	8E-04
rh S oc sup and transversal	3.10	1E-03
rh Pole occipital	-3.09	1E-03
lh G front sup	-3.05	1E-03
lh S front sup	-3.05	1E-03
lh Lat Fis-post	3.02	1E-03
rh G temp sup-Lateral	2.98	2E-03
rh S intrapariet and P trans	2.98	2E-03
lh G orbital	-2.95	2E-03
rh S postcentral	2.94	2E-03
rh S interm prim-Jensen	2.92	2E-03
rh G front sup	-2.92	2E-03
rh G postcentral	2.92	2E-03
rh G and S cingul-Mid-Post	-2.90	2E-03
rh S central	2.90	2E-03
lh S central	2.87	2E-03
lh Lat Fis-ant-Horizont	-2.86	2E-03
rh G and S occipital inf	-2.86	2E-03
rh S circular insula sup	2.86	2E-03
rh S front middle	-2.84	2E-03
rh G occipital middle	-2.84	2E-03
rh S temporal inf	2.83	3E-03
rh G precuneus	-2.83	3E-03
lh G temp sup-Lateral	2.83	3E-03
lh G precentral	2.82	3E-03
lh S orbital med-olfact	-2.79	3E-03
rh G temporal middle	2.78	3E-03
rh G subcallosal	-2.77	3E-03
lh Medial wall	-2.77	3E-03
lh G front inf-Orbital	-2.76	3E-03
lh G front middle	-2.75	3E-03
lh G front inf-Triangul	-2.73	3E-03
rh G front inf-Opercular	2.70	4E-03
lh Pole occipital	-2.68	4E-03
rh Lat Fis-post	2.66	4E-03
rh Pole temporal	2.66	4E-03
lh S calcarine	-2.64	4E-03
lh S temporal sup	-2.60	5E-03
rh S temporal sup	2.59	5E-03
rh S precentral-sup-part	2.56	6E-03
lh G cuneus	-2.54	6E-03
rh G orbital	-2.53	6E-03
rh G precentral	2.53	6E-03
lh S orbital lateral	-2.52	6E-03
lh S front middle	-2.52	6E-03
rh G temp sup-Plan tempo	2.47	7E-03
lh Lat Fis-ant-Vertical	-2.44	8E-03
lh G temporal middle	2.41	8E-03
lh S parieto occipital	-2.40	8E-03
lh G postcentral	-2.37	9E-03

lh G occipital sup	-2.37	9E-03
rh S front sup	-2.35	1E-02
lh G temporal inf	-2.35	1E-02
lh G occipital middle	-2.35	1E-02
rh G and S subcentral	2.34	1E-02
rh G pariet inf-Angular	-2.34	1E-02
lh G precuneus	2.33	1E-02
lh S circular insula ant	-2.32	1E-02
rh G temp sup-Plan polar	2.30	1E-02
lh G insular short	-2.30	1E-02
lh S intrapariet and P trans	-2.30	1E-02
lh G rectus	-2.30	1E-02
lh S postcentral	2.29	1E-02
lh G oc-temp med-Lingual	-2.28	1E-02
rh G parietal sup	-2.27	1E-02
lh S oc middle and Lunatus	-2.26	1E-02
rh G and S cingul-Mid-Ant	-2.25	1E-02
rh S pericallosal	2.25	1E-02
rh G and S paracentral	-2.24	1E-02
rh S subparietal	-2.23	1E-02
rh S parieto occipital	-2.22	1E-02
rh S cingul-Marginalis	-2.21	1E-02
rh S calcarine	-2.21	1E-02
rh G oc-temp med-Parahip	2.21	1E-02
lh G pariet inf-Angular	-2.20	1E-02
rh S orbital-H Shaped	-2.18	2E-02
rh S occipital ant	2.17	2E-02
rh G cingul-Post-dorsal	2.17	2E-02
lh G oc-temp med-Parahip	-2.14	2E-02
lh G and S cingul-Mid-Post	2.13	2E-02
rh G front inf-Triangul	-2.13	2E-02
lh S circular insula sup	2.11	2E-02
rh Lat Fis-ant-Vertical	-2.09	2E-02
lh S suborbital	-2.08	2E-02
rh S circular insula inf	2.08	2E-02
rh G cingul-Post-ventral	2.08	2E-02
lh G temp sup-G T transv	2.06	2E-02
rh G and S transv frontopol	-2.05	2E-02
rh G Ins lg and S cent ins	2.05	2E-02
lh S circular insula inf	2.03	2E-02
rh S temporal transverse	2.02	2E-02
rh G temporal inf	-2.02	2E-02
rh G temp sup-G T transv	2.01	2E-02
lh S oc-temp lat	2.00	2E-02
lh S collat transv post	-1.99	2E-02
lh G temp sup-Plan tempo	1.97	2E-02
lh G Ins lg and S cent ins	-1.96	3E-02
lh S pericallosal	-1.93	3E-02
lh S front inf	1.93	3E-02
lh S precentral-inf-part	-1.92	3E-02

lh G and S frontomargin	-1.92	3E-02
lh G and S transv frontopol	-1.90	3E-02
rh S collat transv ant	1.88	3E-02
lh G and S cingul-Ant	-1.88	3E-02
lh G and S occipital inf	-1.88	3E-02
lh G front inf-Opercular	-1.86	3E-02
lh G and S cingul-Mid-Ant	-1.85	3E-02
lh S oc sup and transversal	-1.84	3E-02
rh S suborbital	-1.83	3E-02
lh S orbital-H Shaped	-1.81	4E-02
rh S oc middle and Lunatus	-1.76	4E-02
rh G cuneus	-1.75	4E-02
lh S temporal inf	1.74	4E-02
lh S temporal transverse	1.73	4E-02
rh S oc-temp med and Lingual	1.72	4E-02
lh S occipital ant	1.71	4E-02
lh S precentral-sup-part	1.69	5E-02
rh S oc-temp lat	-1.67	5E-02
lh S interm prim-Jensen	1.65	5E-02

Supplementary Table S4. Significant differences in NBC between MCI Carriers and non-Carriers groups (FDR-corrected). Values represent the NBC mean and standard deviation (s.d). NBC: Normalize Betweenness Centrality.

Structure	Carriers NBC (s.d)	non-Carriers NBC (s.d)	p-value
Rh G and S cingul-Mid-Post	0.82 (0.17)	1.44 (0.23)	$1.2*10^{-9}$
Rh G oc-temp med-Lingual	1.19 (0.20)	0.94 (0.18)	0.02
Rh Lat Fis-ant-Horizont	0.80 (0.18)	1.36 (0.19)	$0.009*10^{-5}$
Rh S oc-temp med and Lingual	1.46 (0.29)	0.99 (0.19)	0.02
Rh S pericallosal	0.82 (0.18)	1.06 (0.20)	$0.008*10^{-5}$
Rh S temporal transverse	0.80 (0.17)	1.09 (0.17)	$0.007*10^{-3}$
Lh G subcallosal	0.83 (0.20)	1.10 (0.20)	0.04
Lh S circular insula ant	1.08 (0.19)	1.28 (0.23)	0.02
Lh S collat transv post	0.75 (0.15)	1.30 (0.20)	$2.3*10^{-11}$
Lh S precentral-sup-part	0.68 (0.17)	1.10 (0.21)	$0.05*10^{-3}$
Lh S temporal inf	1.19 (0.26)	0.93 (0.19)	0.02

Supplementary Table S5. Hubs regions for Carriers and non-Carriers listing by the descending order of the normalized betweenness centrality in each group

Carriers Hubs	NBC	non-Carriers Hubs	NBC
**Rh S temporal sup**	**2.73**	**Rh G and S cingul-Ant**	**2.69**
**Rh G and S cingul-Mid-Post**	**2.40**	**Rh S parieto occipital**	**2.59**
**Rh G cingul-Post-ventral**	**2.23**	**Lh S parieto occipital**	**2.26**
**Rh Lat Fis-ant-Horizont**	**2.02**	**Lh S oc-temp lat**	**2.15**
**Lh S collat transv post**	**1.81**	**Rh S oc-temp med and Lingual**	**1.99**
**Lh G and S occipital inf**	**1.70**	**Lh G oc-temp med-Lingual**	**1.78**
**Lh S circular insula ant**	**1.65**	**Rh G pariet inf-Supramar**	**1.78**
**Lh G and S subcentral**	**1.59**	**Lh S circular insula sup**	**1.73**
**Rh S collat transv post**	**1.50**	**Lh Lat Fis-ant-Horizont**	**1.69**
Rh G cuneus	1.47	**Rh Lat_Fis-post**	**1.68**
Lh G and S cingul-Ant	1.41	**Lh G and S subcentral**	**1.58**
Rh S front inf	1.35	**Rh S circular insula ant**	**1.57**
Rh G orbital	1.35	**Lh G and S cingul-Ant**	**1.55**
Rh S circular insula inf	1.28	Rh S temporal sup	1.33
Lh G cingul-Post-dorsal	1.19	Lh S occipital _ant	1.31
Rh S orbital med-olfact	1.17	Lh Lat Fis-post	1.22
Lh G temp sup-G T- transv	1.12	Lh S calcarine	1.20
Rh S circular insula ant	1.11	Rh G cingul-Post-dorsal	1.13
Rh S parieto occipital	1.08	Rh S interm prim-Jensen	1.10
Lh S temporal transverse	1.07	Rh G oc-temp med-Lingual	1.07
Lh S front inf	1.04	Rh S orbital-H Shaped	1.07
Lh Lat Fis-post	1.04	Lh G cingul-Post-dorsal	1.03
Rh S front middle	1.03	Rh S collat transv post	1.02
Rh Lat Fis-post	1.03	Rh S precentral-inf-part	1.00

Values represent the regional NBC means. Regions considered hubs per group if NBC>1. In bold hub regions with the higher NBC values (NBC>1.5). NBC: Normalize Betweenness Centrality.

Supplementary Table S6. List of brain regions module composition per group

Structure Full name	Atlas Name	Carriers	non-Carriers
Fronto-marginal gyrus (of Wernicke) and sulcus	G and S frontomargin	V	I

Inferior occipital gyrus (O3) and sulcus	G and S occipital inf	III	II
Paracentral lobule and sulcus	G and S paracentral	II	II
Subcentral gyrus (central operculum) and sulci	G and S subcentral	II (L), IV (R)	I
Transverse frontopolar gyri and sulci	G and S transv frontopo	V	I
Anterior part of the cingulate gyrus and sulcus (ACC)	G and S cingul Ant	V	I
Middle-anterior part of the cingulate gyrus and sulcus (aMCC)	G and S cingul Mid Ant	IV	I
Middle-posterior part of the cingulate gyrus and sulcus (pMCC)	G and S cingul Mid Post	II (L), IV (Ll)	I
Posterior-dorsal part of the cingulate gyrus (dPCC)	G cingul Post dorsal	IV (R), V (L)	III
Posterior-ventral part of the cingulate gyrus (vPCC, isthmus of the cingulate gyrus)	G cingul Post ventral	IV (R), V (L)	III
Cuneus (O6)	G cuneus	III	II
Opercular part of the inferior frontal gyrus	G front inf Opercular	II (L), IV (R)	I
Orbital part of the inferior frontal gyrus	G front inf Orbital	II (L), IV (R)	I
Triangular part of the inferior frontal gyrus	G front inf Triangul	II	I
Middle frontal gyrus (F2)	G front middle	II	II
Superior frontal gyrus (F1)	G front sup	II	II
Long insular gyrus and central sulcus of the insula	G Ins lg and S cent ins	IV	III
Short insular gyri	G insular short	IV	III
Middle occipital gyrus (O2, lateral occipital gyrus)	G occipital middle	III	II
Superior occipital gyrus (O1)	G occipital sup	III	II
Lateral occipito-temporal gyrus (fusiform gyrus, O4-T4)	G oc temp lat fusifor	I	II (R), III (L)
Lingual gyrus, lingual part of the medial occipito-temporal gyrus, (O5)	G oc temp med Lingual	I (R), III (L)	II
Parahippocampal gyrus, parahippocampal part of the medial occipito-temporal gyrus, (T5)	G oc temp med Parahip	I	III
Orbital gyri	G orbital	V	I
Angular gyrus	G pariet inf Angular	III	II
Supramarginal gyrus	G pariet inf Supramar	II	II (R), III (L)
Superior parietal lobule (lateral part of P1)	G parietal sup	III	II
Postcentral gyrus	G postcentral	II	II
Precentral gyrus	G precentral	II	II
Precuneus (medial part of P1)	G precuneus	III	II
Straight gyrus, Gyrus rectus	G rectus	I (L), V (R)	I
Subcallosal area, subcallosal gyrus	G subcallosal	I	I (R), III (L)
Anterior transverse temporal gyrus (of Heschl)	G temp sup G T transv	II	I
Lateral aspect of the superior temporal gyrus	G temp sup Lateral	I	III
Planum polare of the superior temporal gyrus	G temp sup Plan polar	I	III
Planum temporale or temporal plane of the superior temporal gyrus	G temp sup Plan tempo	II	III
Inferior temporal gyrus (T3)	G temporal inf	I	III
Middle temporal gyrus (T2)	G temporal middle	I	III
Horizontal ramus of the anterior segment of the lateral sulcus (or fissure)	Lat Fis ant Horizont	IV (L), V (R)	I
Vertical ramus of the anterior segment of the lateral sulcus (or fissure)	Lat Fis ant Vertical	II (L), IV (L)	I
Posterior ramus (or segment) of the lateral sulcus (or fissure)	Lat Fis post	II (L), IV (R)	I (L), III (R)

Occipital pole	Pole occipital	III	II
Temporal pole	Pole temporal	I	III
Calcarine sulcus	S calcarine	III	II
Central sulcus (Rolando's fissure)	S central	II	II
Marginal branch (or part) of the cingulate sulcus	S cingul Marginalis	II (R), III (L)	II
Anterior segment of the circular sulcus of the insula	S circular insula ant	IV	I
Inferior segment of the circular sulcus of the insula	S circular insula inf	IV	III
Superior segment of the circular sulcus of the insula	S circular insula sup	IV	III
Anterior transverse collateral sulcus	S collat transv ant	I	III
Posterior transverse collateral sulcus	S collat transv post	III	II
Inferior frontal sulcus	S front inf	V	I
Middle frontal sulcus	S front middle	V	I
Superior frontal sulcus	S front sup	II	II
Sulcus intermedius primus (of Jensen)	S interm prim Jensen	III	II
Intraparietal sulcus (interparietal sulcus) and transverse parietal sulci	S intrapariet and P tra	III	II
Middle occipital sulcus and lunatus sulcus	S oc middle and Lunatus	III	II
Superior occipital sulcus and transverse occipital sulcus	S oc sup and transversa	III	II
Anterior occipital sulcus and preoccipital notch (temporo-occipital incisure)	S occipital ant	III	II
Lateral occipito-temporal sulcus	S oc temp lat	I	III
Medial occipito-temporal sulcus (collateral sulcus) and lingual sulcus	S oc temp med and Lingu	I	III
Lateral orbital sulcus	S orbital lateral	V	I (L), III (R)
Medial orbital sulcus (olfactory sulcus)	S orbital med olfact	I (L), V (R)	III
Orbital sulci (H-shaped sulci)	S orbital H Shaped	V	III
Parieto-occipital sulcus (or fissure)	S parieto occipital	III	II
Pericallosal sulcus (S of corpus callosum)	S pericallosal	I (L), V (R)	III
Postcentral sulcus	S postcentral	III	II
Inferior part of the precentral sulcus	S precentral inf part	II (L) , III (R)	I (L), II (R)
Superior part of the precentral sulcus	S precentral sup part	II	II
Suborbital sulcus (sulcus rostrales, supraorbital sulcus)	S suborbital	V	I (L), III (R)
Subparietal sulcus	S subparietal	III	II
Inferior temporal sulcus	S temporal inf	I	III
Superior temporal sulcus (parallel sulcus)	S temporal sup	I (R), III (L)	III
Transverse temporal sulcus	S temporal transverse	I, IV (L)	I

In each structure, the module is identified with roman numbers. R: right hemisphere, L: left hemisphere. G: gyri; S: sulcus.
